# Effects of Vacuum‐Heat‐Assisted Sample Desiccation on Microbiome Surveys

**DOI:** 10.1111/1755-0998.70020

**Published:** 2025-07-28

**Authors:** Stilianos Louca, Claire E. Mullin

**Affiliations:** ^1^ Department of Biology University of Oregon Eugene Oregon USA; ^2^ Institute of Ecology and Evolution University of Oregon Eugene Oregon USA

**Keywords:** metabarcoding, metagenome‐assembled genomes, microbiome, sample desiccation

## Abstract

Sample preservation in the field and during transport can be a logistical challenge for microbiome surveys, particularly in remote areas. Sample desiccation eliminates the need for complicated cold chains and dangerous preservatives. However, the effects of desiccation on modern microbiome workflows such as gene‐centric metagenomic profiling and metagenome‐assembled genome (MAG) binning, remain poorly understood. In addition, most common desiccation tools such as lyophilisation cannot easily be deployed in the field. Here, we describe a proof‐of‐principle sample desiccator using vacuum and heat, specifically built for deployment in the field and exhibiting low power consumption and cost. We then test the effects of vacuum‐heat‐assisted sample desiccation followed by storage at room temperature, in comparison to conventional freezing, on multiple soil and animal faecal samples, via metagenomic and 16S rRNA amplicon sequencing. We consider multiple metrics related to the success of DNA extraction, sequencing, contig assembly, OTU clustering, gene annotation and MAG construction, as well as effects on inferred microbial community composition. We find that the impact of drying on considered success metrics was almost always either minor, non‐significant or positive. For a subset of source materials we observed moderate but statistically significant differences in terms of inferred microbial taxonomic and genetic composition. We conclude that vacuum‐ and heat‐assisted desiccation can be a useful, practical and cost‐effective tool for microbiome field surveys, when a high consistency with frozen samples is not required.

**Abbreviations**
KO: KEGG Ortholog
MAG: metagenome‐assembled genome
OTU: operational taxonomic unit

## 1 Introduction

The realisation that microorganisms strongly impact the functioning of nearly every ecosystem, ranging from the deep ocean and soils to the guts of animal hosts, has led to an intense interest in surveying microbial communities (Thompson et al. 2017). Modern techniques such as metabarcoding (marker gene amplicon sequencing) and metagenomics enable the detection and quantification of millions of microorganisms from a small amount of collected sample material such as wildlife faeces or a teaspoon of soil. However, sample preservation in the field and during transport remains a logistical challenge in microbiome surveys, particularly in remote areas or less developed countries. Cold chains, currently the golden standard of microbial sample preservation, are often impractical or outright impossible in the field. The most common alternative sample preservation methods include (a) preservation in ethanol or other fixatives and (b) desiccation (Hale et al. 2015; Mitchell and Takacs‐Vesbach 2008; Rissanen et al. 2010; Williams et al. 2016). Ethanol and many other fixatives are hazardous materials, which complicate sample storage and shipping, and fixatives tend to substantially increase weight (recommended added quantities typically range within 5–10 sample volumes). Further, fixatives can interfere with downstream molecular analyses and tend to only provide a short‐term storage solution (a few weeks at most), as DNA degradation continues albeit at a slower rate.

Desiccation can be an effective short‐ and long‐term sample preservation method at room temperatures for molecular applications, because in the absence of water most chemical reactions that would otherwise destroy DNA essentially come to a halt. Desiccation greatly facilitates transport in the field, shipping across borders and long‐term archival of samples. Lyophilization (aka. freeze‐drying), in particular, has long been used as a method for preserving viable bacterial cells over multi‐year periods (Bolla et al. 2011; Heckly and Umbreit 1961) and has also been proposed as a means to preserve microbiome samples for sequencing (Bensch et al. 2022; Louca et al. 2016). Although lyophilization is a widely accepted method for biological sample preservation, it requires specialised equipment that is often not available even at institutions in developed countries and is impractical to carry on most expeditions. Approaches using desiccants (e.g., silica) at room temperatures are also problematic, because their slow action increases the risk of sample moulding and DNA degradation before desiccation (Murphy et al. 2000, 2002). Heat‐based or vacuum‐based desiccation, for example using food dehydrators or vacuum desiccators, is commonly used alternatives, but their effects on modern microbiome workflows, including gene‐centric and genome‐resolved metagenomics, are not well understood. Further, the combination of heating and vacuum in the field, which has the potential to reduce drying times and energy consumption, remains to our knowledge largely unexplored. Indeed, a reduction in pressure reduces the boiling temperature of water (Thomson 1946), thus decreasing the energy requirements for sample heating, and reciprocally, warming samples reduces the degree of vacuum that must be maintained.

Here, we examine vacuum‐ and heat‐assisted desiccation as a practical technique for preserving samples collected in the field. Specifically, we present a proof‐of‐principle prototype desiccator using sample heating under vacuum, specifically built for use in expeditions, featuring sufficiently low power consumption for operation and transport in a vehicle. We use this desiccator to compare the effects of desiccation and long‐term storage at room temperature relative to freezing on soil samples from three different locations and faecal samples from three different animals, at various stages of typical microbiome workflows. We emphasise that our objective is not to determine the separate effect of every possible factor such as storage duration, extraction protocol, storage temperature, bioinformatics approaches and so on. Instead, we focus on a few *typical* scenarios that environmental microbiologists can relate to in practice to test the feasibility of vacuum‐ and heat‐assisted desiccation and to determine the extent to which it affects analysis outcomes compared to freezing and relative to other typical sources of error such as sample heterogeneities, stochasticity in PCR and sequencing. We also emphasise that we do not aim to determine whether freezing or drying is more accurate and instead focus on determining the differences between the two. Workflows considered include 16S rRNA amplicon sequencing (aka. metabarcoding) (Thompson et al. 2017), gene‐centric metagenomics for functional profiling (Tringe et al. 2005), and genome‐resolved metagenomics for MAG construction (Parks et al. 2017). We consider 16 different metrics of ‘success’ such as DNA extraction yield, absorbance ratios (representing DNA purity) and peak fragment size, read counts, contig sizes and numbers of recovered operational taxonomic units (OTUs) from amplicons and MAGs from metagenomes. In addition, we examine whether sample desiccation affects inferred microbiome compositions relative to freezing, potentially due to taxonomic biases in either technique.

## 2 Results and Discussion

### 2.1 Sample Desiccation Using Vacuum and Heat

To explore the potential of vacuum‐ and heat‐assisted sample desiccation as a practical tool in the field, we constructed a prototype desiccator using easily available, low‐cost and lightweight materials (photo in Figure S1). We specifically wanted to explore the practical feasibility of such a device built for field work and determine its potential cost and effectiveness. The desiccator is essentially a modified vacuum degassing chamber with added thermostat‐controlled heating blocks for sample heating and an added ceiling heater to restrict condensation of released moisture to the chamber's walls. Vacuum is generated using two serially connected small vacuum pumps. The desiccator can be powered by a 12 V car battery, weighs about 11 kg, and costs about $350 worth of materials to build. In this study, samples were kept at 40°C and < 0.01 bar during desiccation. Further technical details and a step‐by‐step demonstration are given in Data S1 and S2, respectively, and a schematic is given in Figure S2.

To test the effects of such a desiccation approach followed by sample preservation at room temperature on typical microbiome workflows relative to conventional freezing, we collected soil materials from 2 grasslands and 1 forest, as well as faecal materials from 3 different mammals (dog, cat, deer) in a municipal forest. An overview of these materials is given in Figure S3 and Table S1. From each material, we collected five samples to be desiccated and subsequently stored at room temperature, as well as five samples to be frozen at −80°C until further processing. Replication was necessary in order to account for inevitable random effects in sample collection, DNA extraction and sequencing. All samples were kept for 17 months until DNA extraction, followed by 16S rRNA gene amplicon sequencing as well as (shotgun) metagenomic sequencing.

### 2.2 Effects on DNA Extraction

**FIGURE 1:**
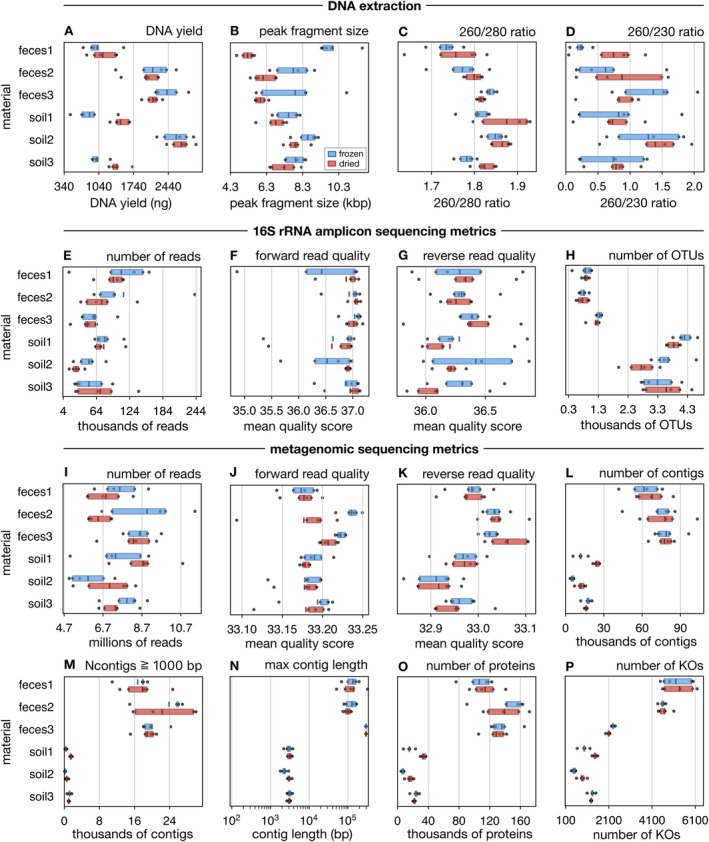
Success metrics by material and treatment. (A) Box‐plots of DNA extraction yields (ng) obtained for each sample (one point per sample), visually grouped by material (faeces 1–3, soils 1–3) and treatment (blue:Frozen, red:Dried). Boxes show interquartile ranges, small vertical bars show means. (B–P) Similar to A, but showing other metrics relevant to DNA extraction (top row), 16S rRNA amplicon sequencing (second row) and metagenomic sequencing, assembly and gene annotation (bottom two rows). For statistical tests quantifying the differences between dried and frozen samples see Table S2.

To examine the effects of sample desiccation relative to freezing on DNA extraction success, we extracted DNA from all samples using the same commercial kit, and measured DNA yield (ng), absorbance ratios at 260/280 nm and 260/230 nm as proxies for purity, as well as peak fragment size (bp). We then compared these four success metrics between the two treatments, whereas controlling for the source material. A visual overview of these metrics, separately for each material and separately for dried and frozen samples, is shown in Figure 1. Although neither dried nor frozen samples achieved a better outcome in all cases, some general tendencies can be recognised. Notably, frozen samples generally yielded larger peak fragment sizes than their dried counterparts, regardless of material (Figure 1B), whereas for almost all materials dried samples yielded a higher average 260/280 ratio than their frozen counterparts (Figure 1C). The drop in peak fragment sizes under drying may be due to additional stress and shear experienced by DNA during desiccation, particularly due to the involved heating (Blake and Delcourt 1998). Results for DNA yields and 260/230 ratios were more ambiguous, with some materials yielding better outcomes when frozen and others yielding better outcomes when dried (Figure 1A,D).

To more quantitatively examine the effects of drying relative to freezing, we also performed statistical location tests for the above metrics using a null model that controls for source material. Specifically, for any given metric we computed its average value for each material and each treatment (dried vs. frozen), computed the ratio of these averages between treatments for each material (i.e., dried average divided by frozen average), then averaged those ratios across all materials, and finally compared that average ratio to that expected under a permutation null model. The null model assumes that for any given material both treatments are statistically equivalent, but allows differences between materials (see Section 2 for details, Table S2 for full results). Importantly, this model does not make any assumptions regarding the distribution of the data such as normality etc. We found moderately strong but significant differences between dried and frozen samples, albeit in mixed directions. Dried samples tended to yield significantly more DNA compared to frozen samples (on average 18% more, *p* = 0.0041, *n* = 60). These differences might be partly due to the higher biomass‐to‐water ratios of dried samples relative to their wet frozen counterparts, combined with the fact that the same start weight was used for all DNA extractions. Future follow‐up studies could normalise extraction inputs by dry weight or include internal spike‐ins to better distinguish true preservation effects from the effects of sample start quantity. Desiccation also tended to significantly reduce the peak fragment size (on average 20% smaller, *p* = 0.0006, *n* = 60). Although for short‐read Illumina sequencing this reduction in peak fragment size is unlikely to be of concern (e.g., the lowest peak fragment size among all samples was still above 4.3 kbp, Figure 1B), it might impact long‐read sequencing such as PacBio or Nanopore. Future studies should thus investigate the compatibility of desiccated samples with long‐read sequencing technologies and resulting metagenomic assembly qualities. Although the observed decrease in DNA fragment size may not significantly affect short‐read Illumina sequencing, future studies should investigate the compatibility of desiccated samples with long‐read sequencing technologies such as PacBio or Nanopore, as well as their impact on metagenomic assembly quality.

A small but significant effect was also observed for 260/280 absorbance ratios, with a 1% higher value on average for dried samples (*p* = 0.015, *n* = 60), whereas no significant difference was observed for the 260/230 absorbance ratio. We point out that 260/280 and 260/230 absorbance ratios may not fully capture the effects of desiccation on DNA purity and quality, for example in terms of inhibitors potentially affecting molecular workflows. We thus also examined the downstream effects in two common molecular sequencing workflows, 16S metabarcoding and metagenomics, discussed in detail below.

### 2.3 Effects on 16S rRNA Amplicon Analyses

To examine the impact of desiccation on typical 16S rRNA amplicon surveys (aka. metabarcoding), we performed 16S rRNA short amplicon paired‐end Illumina sequencing (V4–V5 region) of DNA extracted from all samples. We then compared the number of reads, the average Phred quality score of forward and reverse reads, as well as the number of detected operational taxonomic units (OTUs, at 99% identity), between dried and frozen samples while controlling for source material. These metrics are shown separately for each material and each treatment in Figure 1E–H. Overall, dried and frozen samples yielded comparable outcomes, although some minor differences between treatments can be seen for some materials. Most notably, dried samples yielded on average substantially lower OTUs than their frozen counterparts for soil 1 and 2 (Figure 1H), although for other materials the numbers of OTUs were more similar between treatments. To more quantitatively examine the effects of drying relative to freezing, we also performed statistical location tests for the above metrics using the same null model described in the previous passage (detailed results in Table S2). We found no significant effect of drying compared to freezing in any of the considered metrics. Although on average dried samples yielded 13% fewer reads, 6% fewer OTUs, and 1% lower reverse read qualities compared to frozen samples, none of these differences were statistically significant (*p* > 0.05, *n* = 60).

**FIGURE 2:**
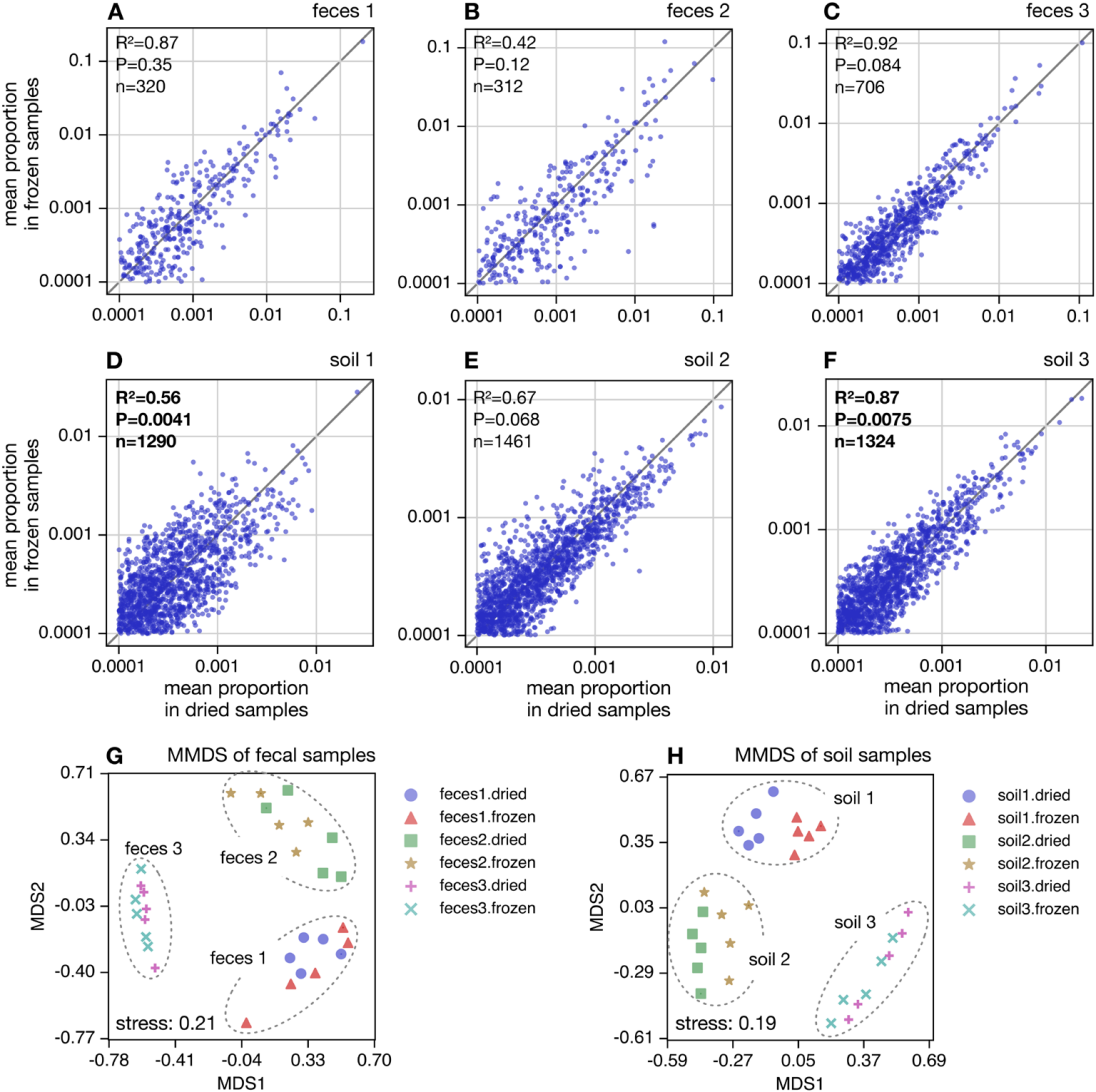
OTU composition vs. treatment. (A) Mean OTU proportions in dried faecal 1 samples (horizontal axis) compared to mean OTU proportions in frozen faecal 1 samples (vertical axis, one point per OTU). Averaging of proportions was done among the 5 replicates in each treatment. The diagonal is shown for reference. Inscriptions show the fraction of variance in the vertical axis explained by the horizontal axis ($R^{2}$), the number of OTUs considered (n), and the statistical significance of $R^{2}$ compared to a permutation null model under which OTU proportions are statistically indistinguishable in the two treatments (P). A significantly low $R^{2}$ (i.e., *p* < 0.05) suggests dried samples tend to yield different OTU proportions compared to frozen samples. (B–F) Similar to A, but for the remaining samples. Statistically significant $R^{2}$ values are boldened. (G) Metric multidimensional scaling (MMDS) plot of weighted Bray‐Curtis dissimilarities between faecal samples, based on OTU proportions. Points correspond to samples, and are shaped and coloured according to the material (faeces 1–3) and treatment (dried vs. frozen). The Kruskal stress is written in the plot. (H) Similar to G, but for soil samples. For similar plots using ASV or genus proportions see Figures S4 and S5, respectively. For MMDS plots based on rarefied data, or based on Jaccard dissimilarities, see Figure S7.

To quantitatively examine how drying affects inferences of microbial community composition relative to freezing, we compared the proportions of OTUs between treatments, separately for each material, as follows. For any given material and any given OTU, we computed the OTU's average proportion across all 5 dried samples and separately across all 5 frozen samples, and then plotted these average proportions on two axes across all OTUs (Figure 2A–F). We measured the agreement between treatments in terms of the fraction of explained variance ($R^{2}$). Overall we observed a strong linear relationship between treatments for all materials, with the strongest agreement seen for faeces 1 and soil 3 ($R^{2}=0.87$) and the lowest agreement seen for faeces 2 ($R^{2}=0.42$), indicating that the two sample preservation methods are largely consistent in broad OTU abundance trends. In the theoretical ideal case of a perfect agreement, all OTUs should have the same average proportion in frozen as in dried samples, that is, all plotted points should be on the diagonal, and $R^{2}$ should be equal to 1. In reality, however, random effects in PCR and sequencing, as well as small microbial content differences between samples collected across a heterogeneous material, lead to variation in OTU proportions between samples, even within the same treatment. In other words, disagreements in average OTU proportions between treatments may or may not be due to the treatment itself. To resolve this ambiguity, we used a statistical test based on a permutation null model that assumes no effects of treatment but controls for material. In this null model, samples from the same material are shuffled randomly regardless of treatment, average OTU proportions are subsequently re‐computed for each treatment, and the $R^{2}$ is re‐computed accordingly. The statistical significance of the observed $R^{2}$ was taken as the probability that the null model would generate a lower $R^{2}$ (i.e., lead to a stronger disagreement between treatments) simply by chance. We found that the $R^{2}$ was significantly low (*p* < 0.05) for 2 out of 6 materials (soil 1 and soil 3), whereas for all other materials $R^{2}$ was non‐significant, that is, the disagreement between samples could plausibly be caused by chance (details in Figure 2A–F). Similar results were obtained at a greater taxonomic resolution of ASVs (roughly representing stains, Figure S4), where soil 1 and 3 were again the sole samples with statistically significant low $R^{2}$ (0.084 and 0.74, respectively). Comparable results were also obtained at the lower taxonomic resolution of genera (Figure S5), where 3 out of 6 materials exhibited a statistically significant low $R^{2}$ (0.91 for faeces 3, 0.77 for soil 1 and 0.92 for soil 3). Such taxonomic differences may be caused, for example, by differential impacts of drying and freeze–thaw cycles on cell preservation and cell release from the material matrix, which in turn can impact the taxonomic distribution of extracted DNA within the relevant fragment size interval.

To assess whether differences in ASV proportions between treatments were due to relative changes among conspecific ASVs (i.e., changes in strain representations within a species) or due to changes at the species level or above, we also performed a modification of the above analysis. Specifically, for each OTU and for each sample, we computed the relative proportions of ASVs within that OTU, that is, such that the sum of ASV proportions in that OTU is 1. We then compared these within‐OTU proportions of ASVs between treatments in the same manner as described in the previous passage (Figure S6). We found no statistically significant low $R^{2}$ for any of the source materials, with $R^{2}$ ranging from 0.83 (faeces 1) to 0.94 (soil 1). This suggests that detection biases mostly occur at the species level or above, and not within a species, in other words most strains within a given species are affected similarly by such biases.

To evaluate the robustness of the above findings, we also considered pairwise abundance‐weighted Bray‐Curtis dissimilarities between samples based on OTU proportions (Legendre and Legendre 1998), and visualised those dissimilarities in the form of 2‐dimensional metric multidimensional scaling (MMDS) plots (Borg and Groenen 2005) separately for faeces and soils (Figure 2G,H). Both for faeces and soils, the three materials are clearly discernible from each other; in other words, samples from each faecal material form a distinct cluster in the MMDS plot, and likewise for soil materials. To confirm the robustness of this observation, we repeated this analysis using abundance‐weighted Jaccard dissimilarities, as well as using rarefied OTU tables in which each sample had the same number of reads (Figure S7). In all cases, we observed a clear and strong separation between materials. Thus, although differences exist between samples in each material, and while these differences may even be partly caused by the treatment, samples from different materials remained clearly distinguishable. In other words, the effects of desiccation—present or not—are much weaker than the differences between examined materials. When narrowing the scope of the MMDS plots to individual materials (Figure S8), for example only comparing dried and frozen samples from faeces 1, a separation between treatments becomes more apparent; however, this separation is generally comparable to the overall spread between samples within a given treatment. Thus, the effects of desiccation on inferred OTU composition seem comparable to those of other typical stochastic factors such as competition during PCR or randomness in the precise location sampled.

To more quantitatively assess if treatment has a detectable effect on pairwise dissimilarities, we used PERMANOVA tests (Anderson 2017), separately for each material and separately for each considered dissimilarity metric. We obtained mixed results depending on the material, the metric considered and the taxonomic level (OTU or genus), detecting a statistically significant separation by treatment in 23 out of 48 cases, mostly among soil materials (Tables S3 and S4). For example, when considering abundance‐weighted Bray–Curtis dissimilarity, we found a significant separation by treatment for 1 out of 6 materials (soil 1) at the OTU as well as genus level. When considering abundance‐weighted Jaccard dissimilarity we found a significant separation by treatment for 3 out of 6 materials (soils 1–3) at the OTU as well as genus level. Hence, desiccation appears to have had a detectable effect on inferred community composition in some materials (mostly soils), but this depended on the considered statistical test and taxonomic resolution. In fact, the fraction of variance in pairwise dissimilarities explained by treatment ($R^{2}$) ranged from 4.6% to 41% at the OTU level and from 3.5% to 58% at the genus level, and was almost always below 50% (Tables S3 and S4). Thus, residual factors other than treatment contributed substantially—typically more than treatment—to stochasticity in community composition.

### 2.4 Effects on Metagenomics

To examine the impact of desiccation on metagenomic surveys, we performed short‐read paired‐end Illumina metagenomic sequencing. We then compared the number of reads, the average Phred quality score of forward and reverse reads, the number of assembled contiguous sequences (contigs), the number of contigs at least 1000 bp long, the maximum contig length, the number of predicted protein‐coding sequences and the number of detected genes (KEGG Orthologs, or KOs), between dried and frozen samples while controlling for material. These metrics are shown separately for each material and each treatment in Figure 1I–P. In general, dried and frozen samples yielded comparable outcomes for these metrics. Some notable exceptions include faeces 3, where dried samples had substantially fewer reads and lower forward quality scores than frozen samples (Figure 1I,J), and soil 1, where dried samples yielded substantially more protein‐coding sequences and KOs than their frozen counterparts (Figure 1O,P). To more quantitatively assess the effects of drying on these metrics, we performed statistical location tests using the same null model as described above (detailed results in Table S2). We found a minor but significant negative effect for forward read qualities (average dried/frozen ratio 0.99, *p* = 0.013, *n* = 60), and strong significant positive effects for the number of contigs (average ratio 1.4, *p* < 0.001, n=60), the number of contigs at least 1000 bp long (average ratio 1.86, *p* < 0.001, n=60), the number of protein‐coding sequences (average ratio 1.41, *p* < 0.001, *n* = 60) and the number of detected KOs (average ratio 1.2, *p* = 0.001, *n* = 60). Hence, in nearly all statistically significant cases drying had a strong positive effect relative to freezing, and in all other cases the effects were either minor and/or insignificant. One reason for the increased number of long contigs in dried samples, and consequently the increased number of detected proteins and KOs, may be that desiccation somewhat lowers the observable species diversity (based on a lower OTU count, see previous section and Table S2), which generally facilitates long contig recovery.

**FIGURE 3:**
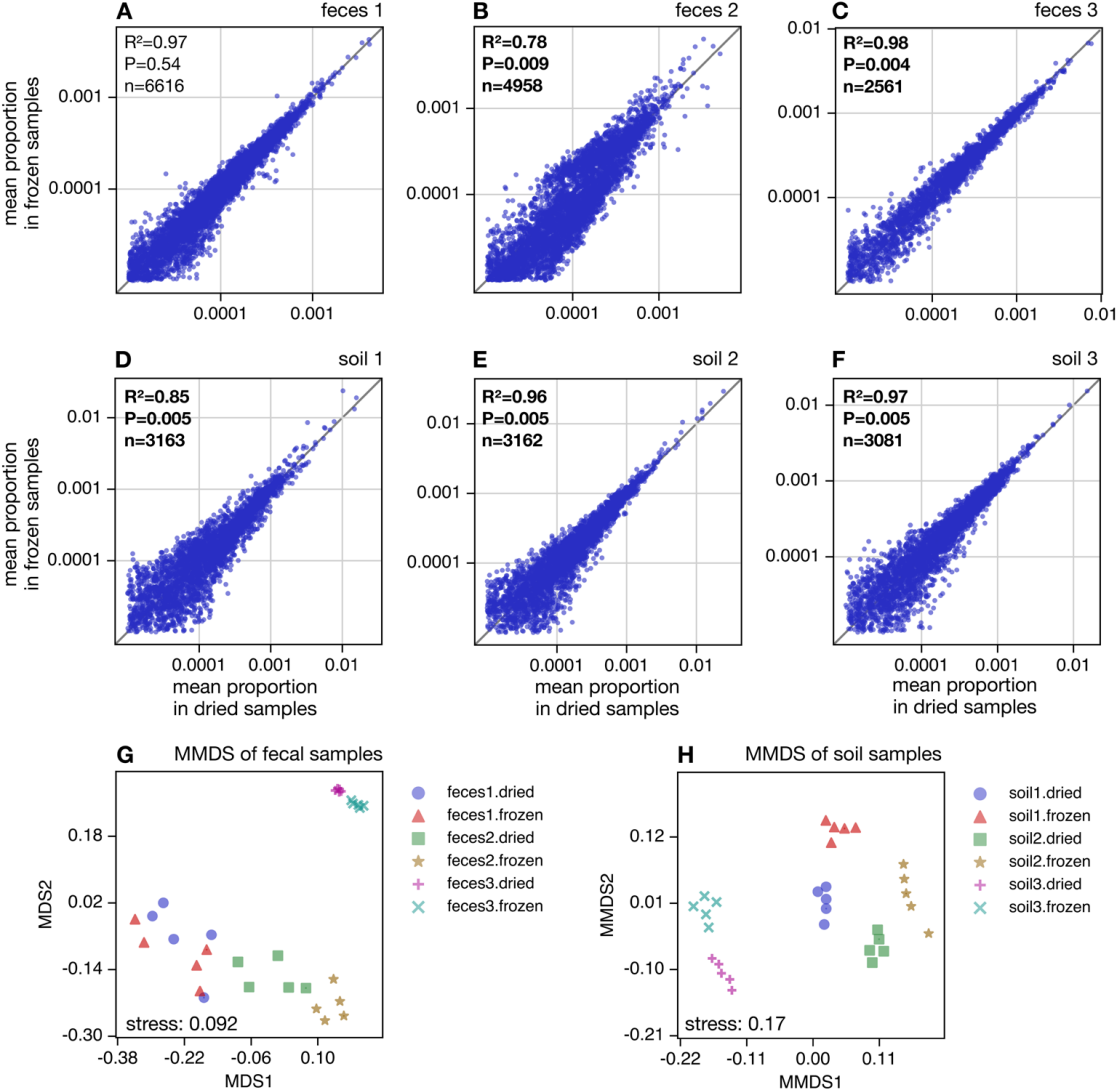
Gene composition vs. treatment. (A) Mean gene (KO) proportions in dried faecal 1 samples (horizontal axis) compared to mean gene proportions in frozen faecal 1 samples (vertical axis, one point per gene). Averaging of proportions was done among the 5 replicates in each treatment. The diagonal is shown for reference. Inscriptions show the fraction of variance in the vertical axis explained by the horizontal axis ($R^{2}$), the number of genes considered (n), and the statistical significance of $R^{2}$ compared to a permutation null model under which gene proportions are statistically indistinguishable in the two treatments (P). A significantly low $R^{2}$ (i.e., *p* < 0.05) suggests dried samples tend to yield different gene proportions compared to frozen samples. (B–F) Similar to A, but for the remaining samples. Statistically significant $R^{2}$ values are boldened. (G) Metric multidimensional scaling plot of abundance‐weighted Bray‐Curtis dissimilarities between faecal samples, based on gene proportions. Points correspond to samples, and are shaped and coloured according to the material (faeces 1–3) and treatment (dried vs. frozen). The Kruskal stress is written in the plot. (H) Similar to G, but for soil samples. For similar plots using gene group proportions at KEGG level C see Figure S10.

To examine how drying affects inferences of the genetic composition of communities (gene‐centric profiles), we compared the proportions of KOs between treatments, separately for each material (KEGG profiles shown in Figure S9). This comparison was done both visually (Figure 3A–F) as well as using a permutation null model, similarly to what we described earlier for OTU proportions. For all materials, we observed a strong linear relationship in average KO proportions between dried and frozen samples, with the fraction of explained variance ($R^{2}$) ranging from 0.78 for faeces 2 up to 0.98 for faeces 3. Hence, drying and freezing indicate similar broad trends in gene abundances. Nevertheless, for 5 out of 6 materials (i.e., except faeces 1) the $R^{2}$ was statistically significantly lower than expected by chance (*p* < 0.05, n=10), indicating that treatment had a detectable impact on inferred genetic composition in these samples. Similar observation were made when KOs were grouped into higher‐level KEGG‐C groups (Figure S10).

To assess the robustness of the above findings, we also considered pairwise abundance‐weighted Bray‐Curtis dissimilarities between samples based on KO proportions (Legendre and Legendre 1998), and visualised those dissimilarities in the form of 2‐dimensional metric multidimensional scaling (MMDS) plots (Figure 3G,H) separately for faeces and soils. Both for faeces and soils, the three materials are discernible from each other, in other words, samples from each material form a distinct cluster in the MMDS plot regardless of treatment, although the separation between materials is not as clear as what we observed for OTUs (Figure 2G,H). To more quantitatively compare dissimilarities between treatments to those within treatments, we performed PERMANOVA tests separately for each material (Tables S5 and S6), similar to what we described earlier for OTU dissimilarities. We found a statistically significant separation between treatments for 5 out of 6 materials (i.e., all except faeces 1, *p* < 0.05, n=10 in each case), both when considering KO profiles (Table S5) or when considering higher‐level KEGG‐C profiles (Table S6). Hence, drying has had a clearly detectable effect on gene‐centric community profiles relative to freezing, for nearly all materials. This conclusion is further confirmed by statistical comparisons of individual KEGG C gene group abundances between treatments, which for nearly all materials (all except faeces 1) revealed several gene groups with statistically significant different proportions even after Bonferroni correction (Table S7, Data S4).

**FIGURE 4:**
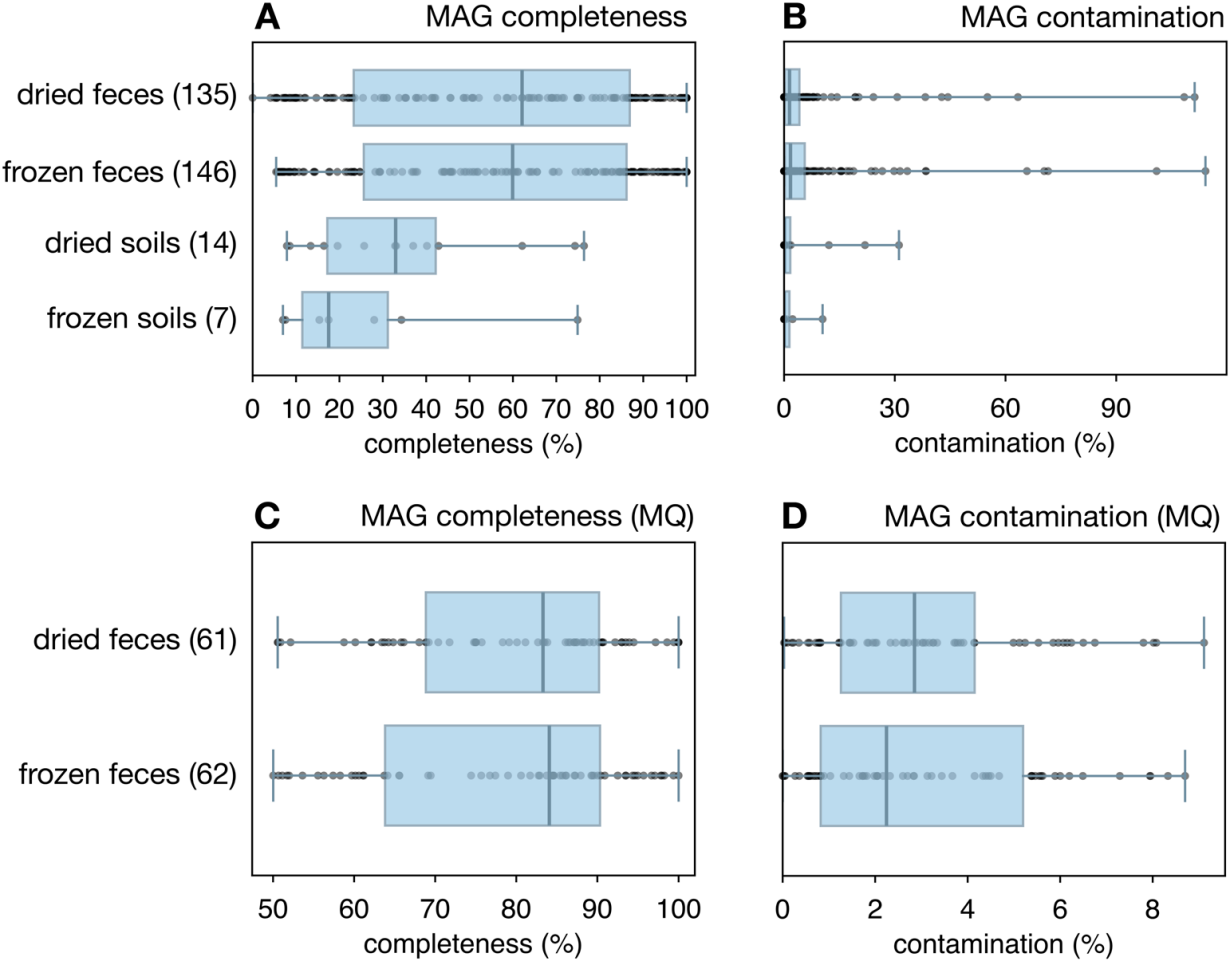
MAG qualities. (A) Box‐whisker plots of MAG completeness levels, separately for each combination of material type and treatment. The number of MAGs is written in the box labels. Boxes show interquartile ranges, whiskers show the full data range, points correspond to individual MAGs, and vertical bars correspond to medians. (B) Similar to A, but showing MAG contamination levels. (C, D) Similar to A, B, but focusing on MAGs of at least medium quality (completeness ≥ 50%, contamination ≤ 10%).

To assess the impact of drying on the ability to construct metagenome‐assembled genomes (MAGs) (Sharon and Banfield 2013), we constructed a separate set of MAGs for each of the following subsets of metagenomes: dried faecal samples, frozen faecal samples, dried soil samples, and frozen soil samples. Splitting our samples in this way allowed us to examine the effects of drying relative to freezing while controlling for the broad material type (faeces vs. soil). Using multiple samples for MAG construction (as opposed to constructing MAGs separately for each sample) allowed us to incorporate differential coverage profiles in the binning process, which is essential for obtaining high‐quality MAGs (Albertsen et al. 2013; Chen et al. 2020). In total 302 MAGs were generated, with 281 and 21 obtained from faecal and soil samples, respectively (overview in Table S8 and Figure 4). The relatively low MAG yield from soil samples is not surprising, as the high microbial diversity found in soils generally makes contig assembly and MAG binning particularly challenging (Ma et al. 2023; Nayfach et al. 2021). For any given material type, the number, completeness and contamination level of MAGs was generally comparable between treatments, with no treatment yielding better results across the board. For example, dried faecal samples yielded slightly fewer MAGs than frozen faecal samples (135 vs. 146), but the situation was reversed for soil samples (14 vs. 7). The mean completeness of MAGs from dried faecal samples was slightly lower than from frozen faecal samples (55.5% vs. 56.9%), but the situation was again reversed for soil samples (35.0% vs. 26.4%). When considering only MAGs of at least medium quality, that is, with completeness ≥ 50% and contamination ≤ 10% (Bowers et al. 2017), we retain 61 MAGs from dried faeces and 62 MAGs from frozen faeces, whereas no MAGs are retained from soils.

### 2.5 Limitations and Future Directions

Our results revealed that sample drying and freezing differ in how they impact the representation of taxonomic and functional groups in the generated sequences. Since both drying and freezing are expected to halt or at least dramatically slow down biological growth, these differences are unlikely to stem from actual microbial community changes, but instead are likely to stem from differential effects on DNA preservation during treatment, storage and DNA extraction. That is, some cell types may resist degradation during desiccation more than others, or may be preserved at room temperature after desiccation better than others, or may be easier to lyse in a desiccated state during DNA extraction than others. Given our data, it is impossible to dissect the contributions of each of these potential processes.

Further, comparative studies such as ours can only determine the effects of sample treatment on microbiome inferences relative to other treatments, but not in absolute terms. In our case, specifically, we determined how vacuum‐ and heat‐assisted desiccation and storage at room temperature affect microbiome inferences relative to freezing, without clarifying whether desiccation or freezing yields the most accurate picture of the true microbial community composition. Indeed, the latter is hard to determine, given the many other biases involved, notably at the DNA extraction and PCR stages (Brooks et al. 2015) and due to variable 16S rRNA gene copy numbers (Louca et al. 2018). In fact, freezing and thawing itself can also introduce taxonomic biases (Cuthbertson et al. 2015; Poulsen et al. 2021).

Finally, we mention that apart from the specific examples examined here, we cannot extrapolate and provide general guidelines on required desiccation times, since these depend not just on initial water content but also on the specific material. Future multifactorial experiments might provide enough data to build a regression model for predicting desiccation times, but until then we think that trial‐and‐error and experience with their own specific types of samples will be the main approach for most researchers.

## 3 Conclusions

We have explored sample desiccation using a combination of vacuum and heat, followed by storage at room temperature, as a practical tool for microbiome field surveys in remote regions. We systematically and quantitatively assessed the effects of sample desiccation on DNA extraction, 16S rRNA amplicon sequencing, and gene‐centric and genome‐resolved metagenomic sequencing, relative to conventional freezing at −80°C. Although our conclusions ultimately remain to be confirmed for other sample types, storage durations and temperatures, DNA extraction protocols, and so on, as well as different levels of microbial diversity, our work provides a demonstration of feasibility and a first assessment of possible impacts of vacuum‐ and heat‐assisted desiccation in the field.

Overall, the observed effects of treatment on success metrics were statistically insignificant or moderate, and either favoured desiccation or freezing depending on the metric. The strongest significant effects were observed in DNA yield and peak DNA fragment size, where dried samples on average performed better and worse, respectively, as well as in the number of contigs, the number of contigs at least 1000 bp long, the number of detected protein‐coding sequences, and the number of detected KOs, in all of which dried samples performed better on average.

For a subset of materials, we observed moderate but statistically significant differences in inferred microbiome compositions, both in terms of taxonomic group proportions and in terms of gene proportions, although samples from different source materials remained clearly distinguishable from each other. In many studies, especially those examining the effects of experimental treatments on microbial communities, it might be more important to maintain consistency in sample collection and processing than trying to infer the precise true composition of microbial communities or to maintain consistency with any particular preservation technique (e.g., freezing). Our results show that in cases where high consistency with freezing is not required, desiccation using vacuum and heat can be a practical sample preservation method in the field.

## 4 Methods

### 4.1 Sample Collection

Surface soil material was collected on 9 November 2022 from three different public locations in the Eugene area including from a municipal forest and two grasslands. Similarly, animal faecal materials were collected on 17 November 2022 around Spencer Butte municipal forest. Based on visual inspection as well as read mapping to reference genomes, faecal materials were identified as originating from dog, domestic cat and mule deer, respectively. An overview of material properties is given in Table S1, photos are provided in Figure S3. All materials were collected in sterile falcon tubes, and were kept at exterior temperatures in their original collection tube for about 1 day until further processing. Each material was split into 2 × 5 samples of roughly 500 mg each, of which 5 were frozen at −80°C until further processing. The remaining five samples were dried in our custom‐built desiccator as described below, and subsequently stored in microcentrifuge tubes inside a carton box at 25°C until further processing. Note that the initial 1‐day exposure to room temperature does not compromise our study's objectives, since it occurred prior to splitting, and thus its effects were shared by all samples from any given material. In fact, as all materials were collected from the natural environment, they had already been experiencing fluctuations in temperature, humidity and other environmental conditions, so one more day at room temperature merely extends that shared history. Replication was necessary in order to account for and quantify typical random effects encountered in the field including heterogeneities in sampled material. In fact, we intentionally did not homogenise the material prior to splitting, in order to fairly represent the inevitable stochasticity in typical field surveys stemming from local material heterogeneities, and to compare that stochasticity with the effects of our treatment (freezing vs. drying). Indeed, one of our goals was to determine how strongly sample preservation impacts scientific outcomes relative to other typical sources of error and bias. Freezing and drying was done on the same day, and thus this time point marks the beginning of divergent treatment between the two sets of samples. In total 60 samples were collected.

Desiccation was done under nearly completely vacuum (< 0.01 bar) at 40°C, using our custom‐built desiccator (described in Data S1, photo in Figure S1, diagram in Figure S2). At this temperature, water boils once the pressure drops below 0.073 bar (Figure S11), which can easily be reached using the desiccator's vacuum pumps. Although a higher temperature could further shorten drying time, it would also increase the risk of DNA damage (Blake and Delcourt 1998). Soil and faecal samples were dried in two separate batches, that is, 30 soil samples were desiccated in one batch and 30 faecal samples desiccated in a separate batch. Samples were desiccated in 5 mL tubes without caps but protected with PTFE filters (pore size 0.22 μm) attached to the tube mouths using Parafilm. The weight loss of samples was periodically estimated using a precision scale and used to assess the progression of desiccation; desiccation was considered complete once the detectable rate of weight loss reached zero. The final fraction of weight lost was taken as an estimate of the material's original water content (% w/w). Soil pH was determined by mixing 20 g of residual soil into 50 mL of deionised water, mixing on a magnetic stirrer for 15 min, letting the slurry rest for another 15 min, then measuring using a West Tune digital pH probe, following common practice (Kirk et al. 2010; Liu et al. 2013). Total desiccation times, pH and water contents are listed in Table S1. In each desiccation batch, we included a negative control consisting of molecular‐grade water in a 5 mL tube (covered with a filter similarly to samples), and placed centrally among the drying samples. Upon desiccation, the controls were rehydrated and vortexed vigorously, DNA was extracted from them as described below and quantified using a Qubit fluorometer and a high‐sensitivity assay; none of the controls yielded any detectable DNA.

In April 2024, that is, about 17 months after sample collection and drying or freezing, all samples were visually inspected for integrity and re‐weighted to test for rehydration. No degradation or notable weight changes were observed, and DNA was then extracted from all samples. This duration was chosen as a representative of typical storage durations encountered in practice, where a considerable time might pass between sample collection and actual sequencing (1–2 years). DNA extractions were performed using the DNeasy PowerSoil Pro kit, following the manufacturer's protocol and always starting with 250 mg of material. Frozen and dried samples from the same material were always extracted on the same day, and all extractions were performed within a period of 1 week. DNA extraction yields were determined using a Qubit 4 fluorometer. Absorbance ratios (260/280 nm an 260/230 nm) were measured using an Implen Nanophotometer NP80. DNA fragment size distributions were measured using an Advanced Analytical fragment analyser. The fragment size at the maximum density, henceforth referred to as ‘peak fragment size’, is considered here as an extraction success metric. We did not quantitatively measure the spread (smear) of the distributions, however upon visual inspection we did not notice any obvious differences between treatments in that regard. The above metrics are visualised using standard box plots in Figure 1, which were generated using the python package matplotlib, functions bxp and scatter.

Shotgun metagenomic and 16S rRNA gene amplicon sequencing was performed for each of the 60 samples by the Integrated Microbiome Resource (IMR) in Dalhousie, Canada. Specifically, metagenomic libraries were prepared using the Illumina Nextera Flex kit and sequenced using a NextSeq2000 (2 × 150 bp paired ends). 16S rRNA gene amplicon fragments (V4–V5 region) were PCR‐amplified using the Phusion Plus polymerase and ‘universal’ bacterial + archaeal primers (515FB = GTGYCAGCMGCCGCGGTAA, 926R = CCGYCAATTYMTTTRAGTTT; Parada et al. 2016; Walters et al. 2015), and sequenced on a MiSeq (2 × 300 bp paired ends).

### 4.2 Statistical Tests of Success Metrics

To statistically examine the differences between dried and frozen samples in each considered success metric (e.g., DNA yield, read qualities etc.), whereas controlling for the source material, we used a permutation‐based location test (Good 2013; Pesarin and Salmaso 2010), as follows. For any given metric, let $X_{m1},..,X_{m5}$ denote the metric's values among the 5 dried samples and $Y_{m1},..,Y_{m5}$ the metric's values among the 5 frozen samples, for the m‐th material (faeces 1–3, soil 1–3). For each material m, we first computed the average value for the dried samples ($X_{m}≔\left(X_{m1}+..+X_{m5}\right)/5$) and separately for the frozen samples ($Y_{m}=\left(Y_{m1}+..+Y_{m5}\right)/5$). We then compared these average values by forming their ratio for each material, $\rho_{m}≔X_{m}/Y_{m}$. Hence, for example, a ratio $\rho_{m}>1$ means that drying had an overall positive effect in the considered metric among samples of the m‐th material. We then computed the average of these ratios, $\rho ≔\left(\rho_{1}+..+\rho_{6}\right)/6$ in order to quantify the overall effect of drying relative to freezing across all materials (Table S2). To assess the statistical significance of this ratio ρ, we compared it to ratios expected under a permutation null model that assumes no statistical differences between frozen and dried samples but still controls for the material. The null model randomly permutes the samples within each material, thus breaking any associations between outcome and treatment but maintaining associations between outcome and material. We performed 10,000 such permutations, and for each permutation the $X_{m}$, $Y_{m}$, $\rho_{m}$ and ρ were re‐computed, thus yielding an empirical distribution of ρ under the null model. The statistical significance of the observed ρ was taken as the fraction of ρ generated by the null model that were at least as extreme as observed in either direction, that is, with an absolute distance from 1 at least as large as observed. One of the advantages of the above permutation approach, compared to for example conventional linear regression, is that we do not need to make any assumptions about the probability distribution of the data (Gaussian or not, constant variances or not; Good 2013). Further, it allowed us to conveniently implement a null model that directly controls for source material while assessing the statistical significance of differences between treatments.

### 4.3 Analysis of 16S rRNA Gene Amplicons

On average 71,036 16S rRNA gene read pairs were obtained for each sample. Reads were quality‐filtered and amplicon sequence variants (ASVs) were inferred and chimera‐filtered using the R package dada2 v1.28.0 (Callahan et al. 2016), as follows. Reads were quality‐filtered using the dada2 function filterAndTrim, with options ‘truncLen = (250,200), maxEE=(1,1), truncQ=(0,0), trimLeft=(6,6), minLen = (100,100), maxLen = (100000,100000)’, retaining on average 60,076 read pairs per sample. Error model calibration for ASV inference was performed jointly for all samples but separately for forward and reverse reads. Calibration was performed using the dada2 function learnErrors with options ‘nbases=1e8, randomize=TRUE, MAX_CONSIST=10, errorEstimationFunction=loessErrfun’. Reads were dereplicated using the dada2 function derepFastq. ASVs were inferred from dereplicated sequences separately for forward and reverse reads, using the dada2 function dada (options ‘pool=TRUE, selfConsist=FALSE’) and the previously calibrated error models. ASVs from forward and reverse reads were merged using the dada2 function mergePairs with options ‘minOverlap=12, maxMismatch=0, trimOverhang=TRUE’. Merged ASVs were chimera‐filtered using the dada2 function removeBimeraDenovo (option method = ‘consensus’). ASVs were taxonomically classified based on a comparison to the SILVA database v138.1 (Glöckner et al. 2017), using a consensus approach (Soufi, Tran, and Louca 2024). Non‐prokaryotic ASVs, including mitochondria and chloroplasts, were omitted from all subsequent analyses. Retained ASVs were then clustered de‐novo into operational taxonomic units (OTUs) at 99% similarity, a commonly used species delineation threshold (Edgar 2018; Kim et al. 2014), using vsearch –cluster_fast with options ‘–iddef 2 –strand plus’. This yielded an OTU table with 17,680 OTUs representing 2,126,128 reads across 60 samples.

### 4.4 Statistical Comparisons of Microbial Taxonomic Compositions

Pairwise dissimilarities between samples in terms of microbial taxonomic composition (OTU and genus level) were computed using two alternative popular metrics, abundance‐weighted Bray‐Curtis and abundance‐weighted Jaccard, both of which account for relative OTU/genus abundances (Chao et al. 2005; Legendre and Legendre 1998). We computed these metrics using the full data as well as using a rarefied subset where all samples were subsampled to the same number of reads. Metric multidimensional scaling (MMDS) embedding into 2 dimensions based on these pairwise dissimilarities was performed by minimising the Kruskal stress function (Borg and Groenen 2005) (Figure 2G,H, Figures S7 and S8). Principal Coordinates Analysis (PCoA) was also performed for comparison, shown in Figure S12 and confirming the separation between materials seen in the MMDS plots.

To test whether drying or freezing had any effects on inferred OTU or genus compositions, PERMANOVA tests were performed on these dissimilarities, using the pseudo *F* test statistic and estimating statistical significances via 10,000 permutations (Anderson 2017). A significantly high pseudo *F* statistic indicates that pairwise dissimilarities between treatments tend to be detectably larger than within each treatment (Tables S3 and S4).

To further visualise and quantify potential differences in OTU composition between treatments for any given material, we computed for each OTU its average proportion across all 5 dried samples (denoted $X_{i}$) and its average proportion across all 5 frozen samples (denoted $Y_{i}$), and then visually compared these average proportions across OTUs using the python function pyplot.scatter (Figure 2A–F). In addition, we computed for each material the fraction of variance in the $Y_{i}$ explained by $X_{i}$, also known as coefficient of determination:

(1) $$R^{2}=1-\frac{\sum_{i}{\left(Y_{i}-X_{i}\right)}^{2}}{\sum_{i}{\left(Y_{i}-\bar{Y}\right)}^{2}}$$

where i iterates over all OTUs and $\bar{Y}$ is the average of $Y_{1},Y_{2},\ldots$. A lower $R^{2}$ indicates a stronger disagreement between frozen and dried samples. The statistical significance of $R^{2}$ was estimated based on a permutation null model in which frozen and dried samples are statistically indistinguishable. In this null model, the treatments (frozen vs. dried) associated with samples were randomly permuted, thus breaking any statistical association between samples and treatment. For each permutation, average OTU proportions in each treatment and the corresponding $R^{2}$ were re‐computed, and the statistical significance (*p*‐value) was set to the fraction of all 10,000 permutations that yielded a lower $R^{2}$ than observed. Only OTUs with a mean proportion of at least 0.01% in both treatments were considered in this analysis, to avoid excessive noise associated with sampling stochasticity. A similar approach was used to also compare ASV proportions (Figure S4) and genus proportions (Figure S5) between treatments.

To assess whether differences in ASV proportions between treatments were due to relative changes among conspecific ASVs or due to changes at the species level, we proceeded as follows. For each OTU and for each sample, we computed the relative proportions of ASVs within that OTU, that is, such that the sum of ASV proportions in that OTU is 1. Only ASVs also included in the previous analysis were considered (i.e., representing at least 0.01% of reads in a sample) to ensure comparability. We then compared these within‐OTU proportions of ASVs between treatments in the same manner as described earlier for the within‐sample proportions (Figure S6).

### 4.5 Analysis of Metagenomes

On average 7,586,082 metagenomic read pairs were obtained per sample. Adapters were trimmed from reads using the tool skewer v0.2.2 (Jiang et al. 2014). Reads were then quality‐filtered using vsearch v2.28.1 (Rognes et al. 2016) with options “–fastq_ascii 33 –fastq_maxee 1 –fastq_truncee 1 –fastq_qmax 64 –fastq_maxee_rate 0.005 –fastq_stripleft 0 –fastq_trunclen_keep 10000”, retaining on average 5,997,909 high‐quality read pairs per sample. Paired reads from each sample were assembled into longer contiguous sequences (contigs) using megahit v1.2.9 (Li et al. 2015) with option “–min‐contig‐len 500”. On average 43,527 contigs were generated per sample. Gene‐centric functional profiles were generated from assembled contigs similar to (Soufi, Porch, et al. 2024; Soufi, Tran, and Louca 2024). Here we thus only provide a brief summary. Contig coverages were computed for each sample by mapping the non‐assembled reads to the contigs, then counting the number of reads mapped to each contig with a MAPQ score ≥ 30 and dividing that number by the contig length. Contig coverages were then normalised in each sample to sum 1, thus yielding contig “proportions”. Protein‐coding genes (PCGs) were predicted in the contigs using prodigal v2.6.3 with option “‐p meta” and otherwise default options (Hyatt et al. 2010). PCGs were then mapped to KEGG gene orthologs (KOs) in the KOfam HMM database r105 (Aramaki et al. 2019) using hmmsearch v3.3 (Mistry et al. 2013). Only hits with an E‐value below $10^{-10}$ were considered.

Gene‐centric functional profiles were estimated for each sample as follows. Quality‐filtered paired reads from multiple samples were coassembled separately for each combination of treatment and material type (i.e., dried faeces, frozen faeces, dried soils, frozen soils). Contig coverages in each sample were computed and PCGs were predicted as described earlier. Proportions of PCGs were computed in each sample by first associating with each PCG the proportion of its host contig, and then normalising those values in each sample to sum 1. The proportion of a given gene in a given sample was estimated by summing the proportions of all PCGs mapped to the gene. KOs were also grouped into standard high‐level functional groups at KEGG level C; the proportion of each group in each sample was computed as the sum of proportions of all associated genes (Figure S9). The following KEGG groups were omitted, as they are not actually defined based on function: ‘brite hierarchies’, ‘enzymes with ec numbers’, ‘not included in pathway or brite’, ‘poorly characterised’, ‘general function prediction only’, ‘others’, ‘unclassified viral proteins’, ‘function unknown’.

Metagenome‐assembled genomes (MAGs) were constructed from the coassembled contigs as follows. Note that coassembling across multiple samples allowed us to use differential contig coverages to improve MAG binning (Albertsen et al. 2013; Chen et al. 2020). For each sample, reads were mapped back to contigs using bowtie2 v2.5.1 (Langmead and Salzberg 2012) with option ‘–no‐unal’. SAM files generated by bowtie2 were converted to BAM files using samtools v1.17. BAM files and contig fasta files were used as input to MetaBAT2 v2.15‐2 (Kang et al. 2019) for binning contigs into MAGs, with options ‘‐m 3000 ‐s 200000’. This yielded a separate set of MAGs for each combination of treatment and material type. MAG qualities (completeness & contamination) were estimated using checkM2 v1.0.1 (Chklovski et al. 2023). MAGs were taxonomically identified using the GTDB‐Tk toolkit, workflow classify_wf v2.3.0 (Chaumeil et al. 2020). An overview of generated MAGs is given in Table S8. MAG completeness and contamination levels are visualised using standard box plots in Figure 4, which was generated using the python package matplotlib, functions bxp and scatter.

### 4.6 Statistical Comparisons of Microbial Gene Composition

Pairwise dissimilarities in terms of gene (KO) or gene group (KEGG C) composition were computed using the abundance‐weighted Bray‐Curtis metric (Legendre and Legendre 1998). Metric multidimensional scaling and PERMANOVA analyses based on those dissimilarities were performed as described earlier for OTUs. Statistical comparisons of KEGG C gene group abundances between treatments (location test), separately for each material, were conducted using the Welch test statistic (Alekseyenko 2016) and a permutation null model (Table S7, Data S4). Further, gene and gene group proportions were visually and statistically compared between treatments using a similar approach as described above for OTUs (Figure 3). Only genes with a mean proportion of at least 0.001% in both treatments were considered in this analysis to avoid excessive noise associated with sampling stochasticity.

## Author Contributions

S.L. designed the study, built the prototype desiccator, performed the sample desiccations and data analyses, and wrote a first version of the manuscript. C.E.M. performed the molecular work and contributed to the writing of the manuscript.

## Disclosure

Benefit‐sharing statement: Benefits from this research result from potential technological and methodological innovations inspired by our study, as well as the sharing of our data as described above.

## Conflicts of Interest

The authors declare no conflicts of interest.

## Supporting information


**Appendix S1:** men70020‐sup‐0001‐AppendixS1.pdf.

**Data S1:** men70020‐sup‐0002‐DataS1.tsv.

**Data S2:** men70020‐sup‐0003‐DataS2.tsv.

**Data S3:** men70020‐sup‐0004‐DataS3.tsv.

**Data S4:** men70020‐sup‐0005‐DataS4.xlsx.

## Data Availability

Raw metagenomic and amplicon sequence data are available on the NCBI Sequence Read Archive under BioProject PRJNA1199176, BioSamples SAMN45866904–SAMN45866963, runs SRR31751350–SRR31751409 (metagenomes) and SRR31749508–SRR31749567 (16S rRNA gene amplicons). Sample metadata with 16S rRNA success metrics are available as Data S1. Sample metadata with metagenomic success metrics are available as Data S2. MAG details, including completeness, contamination, and taxonomic identity, are given in Data S3. Statistical comparison results (location tests) for individual KEGG‐C group proportions are given in Data S4. Other intermediate data outputs, such as contigs or OTU representative sequences, are available from the corresponding author on reasonable request. All software used in this paper has been described in Section 2 and are freely available online.
