# Supplementary material for: Effects of Vacuum‐Heat‐Assisted Sample Desiccation on Microbiome Surveys
Source: Mol Ecol Resour. 2025 Jul 28;25(7):e70020. doi: 10.1111/1755-0998.70020 (PMC12415838; doi:10.1111/1755-0998.70020)
Supplement: Supplementary file 1 — Appendix S1: men70020‐sup‐0001‐AppendixS1.pdf. [file MEN-25-e70020-s003.pdf]

Effects of vacuum-heat-assisted sample desiccation on microbiome surveys
# - Supplemental Materials -

Stilianos Louca[1,2,*] & Claire E. Mullin[1,2]

[1]Department of Biology, University of Oregon, Eugene, USA
[2]Institute of Ecology and Evolution, University of Oregon, Eugene, USA
[*]Corresponding author

S.1 Description of desiccator

In this section we describe the vacuum-heat-assisted desiccator built and used in this study (photo in Fig. S1, diagram in Fig. S2). We hope that the details provided will help others replicate or further improve upon our prototype for their own purposes. The desiccator consists of a vacuum chamber with internal temperature-controlled electric heating. The vacuum chamber is a modified cylindrical 1.5 gallon BACOENG™ vacuum degassing chamber with stainless steel walls, glass lid, silicone sealing gasket and pre-attached vacuum gauge. Vacuum is established using two small serially connected oilless diaphragm vacuum pumps (brand Maisi, model TC-150C, max flow 15 L/min), which combined can achieve nearly complete vacuum (<0.01 bar) in less than 5 minutes. Heating of samples is done in two conventional rectangular dry bath aluminum heating blocks, which can accommodate up to 24 centrifuge tubes of 5 mL capacity. Each block is heated using two ceramic Positive Temperature Coefficient (PTC) elements, with a terminal temperature of 60°C at 12 V under standard conditions. To allow for arbitrary target temperatures, the PTC elements of each block are powered through a digital adjustable temperature controller, with the controller's temperature sensor being inserted in the block center. In our case, each controllers' target temperature was set between 40°C (low) and 41°C (high). At this temperature, water begins to boil when the chamber's pressure drops below 0.073 bar or 55.12 mm Hg (Fig. S11). If desiccation is to be performed at lower temperatures (for example to reduce power consumption), a greater vacuum is needed.

Water removal: The water released by the drying samples is partly removed through the pumping progress, however some residual moisture remains and condensates inside the chamber. To prevent condensation on the glass ceiling, which would risk water dropping back into the open sample tubes, we also attached two ceramic PTC heating elements on the ceiling's interior surface. Heating the ceiling ensures that condensation is restricted to the colder steel walls of the chamber. Water droplets forming on the walls ultimately descend to the chamber's metallic bottom, where they can be wiped off with a cloth once desiccation is complete. The ceiling PTC elements have a terminal temperature of 70°C at 12V, although PTCs with a lower terminal temperature would probably suffice as well.

Power consumption: The desiccator is powered by 12V DC, which is available in most personal vehicles or may be delivered by a deep-cycle battery. Under standard conditions, the maximum power consumption

is 40 W for the two pumps, 40W for the four sample-heating PTC elements, and 77 W for the ceiling PTC elements. The PTC elements' power consumption rapidly decreases as their temperature increases. At around 40°C, the sample-heating PTC elements consume only 20 W (when active) and the ceiling PTC elements consume only 18 W. Similarly, the power consumption of the pumps decreases as vacuum is established, and near vacuum the pumps consume only 27 W. Thus, overall power consumption gradually drops during the desiccator's startup. Each of these three parts (block PTCs, ceiling PTCs, pumps) can be powered on and off independently, to limit the initial peak power consumption if necessary. Hence, for example, one may first establish the vacuum, then turn the pumps off, activate the ceiling heating until power consumption drops, and lastly activate the sample heating. Based on our experience, the vacuum pumps only need to be run for a few minutes every 2–3 hours to maintain adequate vacuum throughout the desiccation process. Once the target temperature of 40°C is reached, the long-term average power consumption to needed maintain this temperature is 21 W. Hence, over a 24 hour operation the prototype consumes approximately 0.5 kWh.

Wiring: All necessary wires entering the vacuum chamber, i.e., for powering the PTC elements and for the temperature sensors, are driven through small holes drilled into the steel walls and sealed using marine-grade adhesive sealant. It was important to also seal the ends of all wires to prevent air from leaking into the vacuum chamber and eroding the vacuum. Indeed, during early trials we observed that air could enter some open-ended wires at the ends and be sucked through those wires into the chamber.

Size and cost: The desiccator is mounted in a sturdy plastic Hudson Exchange™ container, with dimensions $24\times15\times11$ inches and partly padded with a rubber mat for sound insulation. The desiccator's total weight, including sound insulation material and carrying case, is about 11 kg. The total cost of construction materials was around $350 USD in 2022; industrial production of such a device at higher quantities is expected to push this cost down significantly.

Operational stability: During our sample desiccations, vacuum pressure varied slightly during the first 5 minutes of the desiccation phase due to the release of relatively large amounts of vapor from the samples, but never exceeded 0.01 bar. Temperature distribution inside the vacuum chamber was assessed using two thermocouples spaced 10 cm apart, with reading differences between them generally being below 1°C except for the initial warm-up phase (up to 3°C difference in the first 10 minutes) due to slight manufacturing differences between nominally identical heating elements. While we do not consider these heterogeneities to be of major importance in most practical applications, if desired one might achieve improvements using higher quality heating elements and more efficient vacuum pumps.

Major envisioned improvements: Based on our experience, we can particularly recommend the following future improvements:

- Install an internal scale for continuously measuring weights and thus monitoring desiccation progress without the need to open the chamber.
- Increase the thermal insulation of the chamber in order to reduce power consumption.
- Use PTCs with a lower terminal temperature to heat the chamber's ceiling, to reduce power consumption.
- Increase the sample capacity relative to the device's footprint by using heating blocks shaped to fit and occupy the full chamber.
- Use wider and shallower sample wells and tubes to increase active evaporation surface and accelerate desiccation.

S.2 Usage of desiccator

This section demonstrates the use of our desiccator step by step. We start with two soil samples collected into sterile 5 mL centrifuge tubes, one from a muddy puddle (S1) and one from a dry garden lawn (S2).

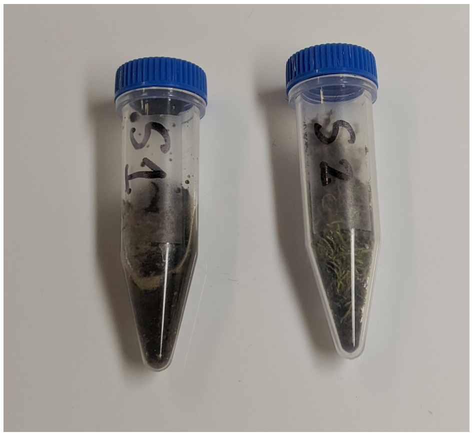

Remove the cap of each sample tube, cover the opening with a PTFE or PES filter membrane (pore size 0.2 μm, diameter 2-3 cm), firmly held in place using some parafilm™. Ensure that the parafilm fully seals the gap along the mouth's perimeter, while minimizing the amount of parafilm covering the mouth so as to allow the escape of vapor.

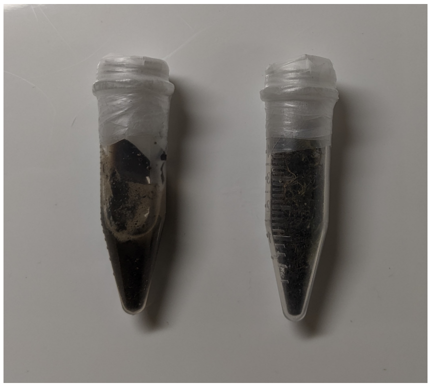

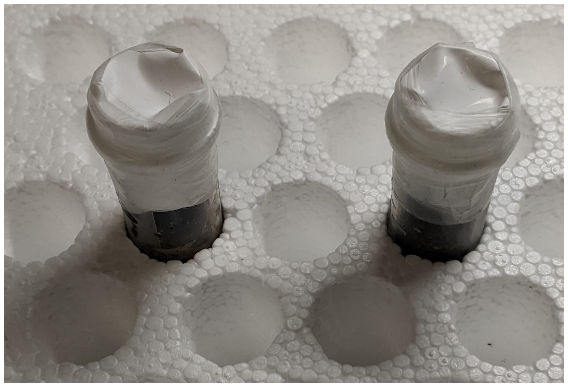

Place the sample tubes into the heating blocks inside the desiccator's vacuum chamber. Also ensure that the temperature sensors are inserted into the proper slot in the heating blocks.

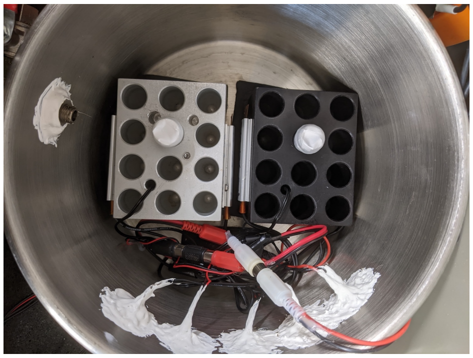

Cover the vacuum chamber with its lid, ensuring that the lid is centered to avoid any gaps. Close the vacuum chamber's vent valve and open its connection valve to the pump.

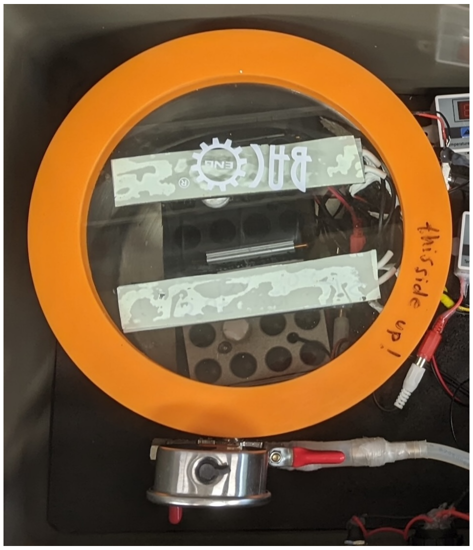

Note that our desiccator has two PTC heating elements glued to the lid's interior, visible above, to prevent water condensation on the lid during desiccation. Next, connect the desiccator to a 12V DC power supply, for example a portable deep cycle battery, a car's "cigarette lighter" power supply, or through an AC-DC converter to a residential power outlet. Our desiccator uses a female barrel jack (5.5×2.1 mm) for power input. Please see Supplement S.1 regarding the desiccator's adjustable power consumption.

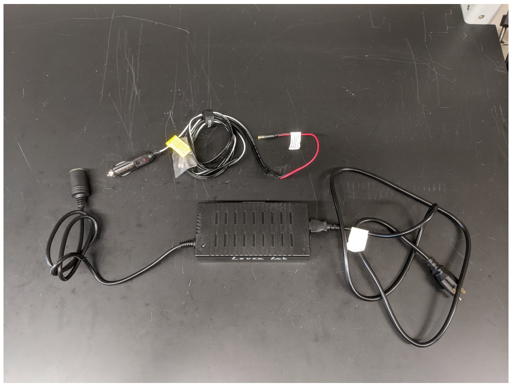

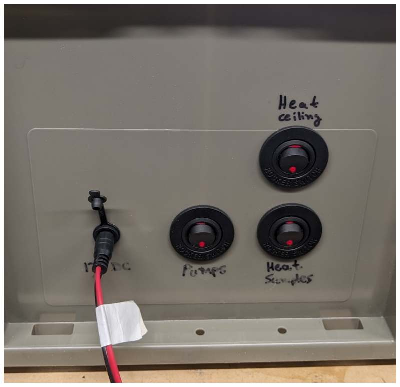

The following electric activation sequence is designed to reduce peak power consumption. Turn on the ceiling heaters and wait for 10 minutes until they approach their terminal temperature, at which point their power consumption will be substantially reduced. Then turn on the thermostat-controlled PTC elements on the heating blocks, ensuring that each thermostat's target temperature range is 40–41°C. The current temperature of the heating blocks can be seen on the thermostat displays.

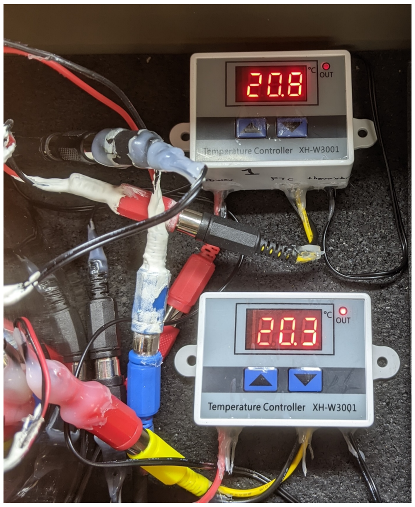

Once the target temperature has been reached for both heat blocks, turn on the vacuum pumps. Wait for a few minutes until nearly complete vacuum is reached and pressure has stabilized (<0.01 bar). The rate of vapor release from the samples will now typically be at its highest, and will gradually decrease thereafter. Some of the vapor will condensate on the chamber's walls, and some of it will be removed via the pumps. Close the lid of the desiccator's container to minimize noise from the pumps.

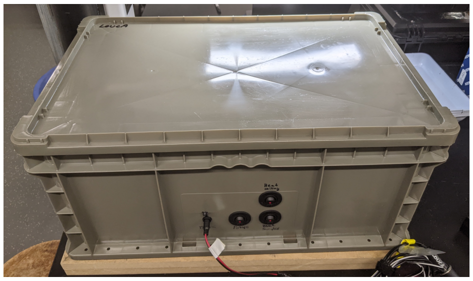

From this point forward you may keep the vacuum pumps running, or you may turn them off and only turn them on occasionally briefly to restore vacuum as necessary. If you have more samples, or samples with higher water content, more frequent pumping will be necessary. Further, if you plan to occasionally weigh the samples to monitor desiccation progress, you will need to restore vacuum as well. For this demonstration we kept samples in the desiccator for a predetermined 12 hours. Repeat pumping was only necessary after an initial 3 hours, at which point pressure had risen to about 0.03 bar. The dried samples are shown below:

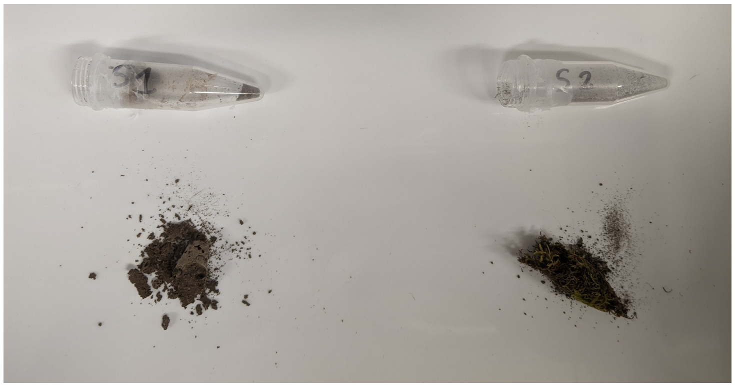

Table S1: **Materials overview.** Overview of materials considered. Note that from each material we collected 2×5 samples, of which 5 were frozen and 5 were dried.

**Material**	**description**	**pH**	**water content (% w/w)**	**hours dried**
feces 1	dog feces	NA	81.9%	21.05
feces 2	feline feces	NA	68.8%	21.05
feces 3	mule deer feces	NA	48.1%	21.05
soil 1	surface soil, urban forest	6.59	18.1%	7.55
soil 2	surface soil, urban grassland	6.86	18.7%	7.55
soil 3	surface soil, urban park	7.90	23.1%	7.55

Table S2: **Statistical comparisons of success metrics.** Overview of location tests of various success metrics, including the average ratio of dried over frozen (averaged over all materials), standardized effect size (SES) and two-sided statistical significance (P-value). P-values below 0.05 are bolded. All tests are based on 60 samples, and P-values are estimated using a permutation null model described in the main article.

**Metric**	**Average ratio dried/frozen**	**SES**	**P-value**
DNA extraction			
DNA yield (ng)	1.18	+3.0	**0.0041**
peak fragment size (bp)	0.80	-3.82	**0.0006**
260/280 ratio (nm/nm)	1.01	+2.42	**0.0146**
260/230 ratio (nm/nm)	1.37	+1.79	0.10
16S rRNA amplicons			
number of reads	0.87	-0.89	0.39
forward read quality	1.01	+1.41	0.16
reverse read quality	0.99	-1.22	0.22
number of OTUs (α-diversity)	0.94	-1.36	0.18
metagenomes			
forward read quality	0.99	-2.37	**0.013**
reverse read quality	1.00	+0.64	0.53
number of reads	0.98	-0.38	0.71
number of contigs	1.40	+4.93	**<0.001**
number of contigs $\geq$1000 bp	1.86	+6.18	**<0.001**
max contig length (bp)	1.04	+0.50	0.62
number of proteins	1.41	+4.84	**<0.001**
number of KOs	1.20	+4.02	**0.0001**

Table S3: **PERMANOVA analysis, OTU composition.** PERMANOVA test results, comparing various dissimilarity metrics in OTU composition between dried and frozen samples, separately for each material. Considered metrics include abundance-weighted Bray-Curtis with and without rarefaction and abundance-weighted Jaccard with and without rarefaction. Shown are the pseudo F-statistic, the standardized effect size (SES), the statistical significance (P) and the fraction of variance in pairwise distances explained by the treatment (R^2). A P value below 0.05 (shown in bold) indicates systematic differences in inferred OTU composition between dried and frozen samples. Each test is based on 10 samples (5 dried, 5 frozen).

**Material**	**F-statistic**	**SES**	**P**	R^2
Bray-Curtis, non-rarefied				
feces 1	0.85	-0.36	0.57	0.046
feces 2	1.66	1.19	0.11	0.13
feces 3	1.09	0.041	0.28	0.075
soil 1	4.81	6.10	**0.0082**	0.41
soil 2	1.92	1.62	0.064	0.15
soil 3	1.10	0.011	0.25	0.068
Bray-Curtis, rarefied				
feces 1	1.04	-0.14	0.41	0.074
feces 2	1.89	1.19	0.090	0.14
feces 3	2.39	3.22	**0.0077**	0.23
soil 1	3.67	6.54	**0.0081**	0.37
soil 2	1.49	2.11	**0.044**	0.15
soil 3	1.87	4.73	**0.0044**	0.20
Jaccard, non-rarefied				
feces 1	0.089	-0.67	0.69	0.0083
feces 2	1.12	0.043	0.36	0.076
feces 3	1.46	1.35	0.12	0.093
soil 1	4.64	6.39	**0.0031**	0.39
soil 2	2.39	2.92	**0.026**	0.16
soil 3	1.84	3.66	**0.0017**	0.11
Jaccard, rarefied				
feces 1	0.59	-0.52	0.68	0.037
feces 2	2.29	1.06	0.12	0.10
feces 3	1.58	2.61	**0.013**	0.16
soil 1	2.75	6.19	**0.0006**	0.29
soil 2	1.47	2.62	**0.029**	0.15
soil 3	1.51	5.28	**0.0079**	0.16

Table S4: **PERMANOVA analysis, genus composition.** PERMANOVA test results, comparing various dissimilarity metrics in genus composition between dried and frozen samples, separately for each material. Considered metrics include abundance-weighted Bray-Curtis with and without rarefaction and abundance-weighted Jaccard with and without rarefaction. Shown are the pseudo F-statistic, the standardized effect size (SES), the statistical significance (P) and the fraction of variance in pairwise distances explained by the treatment (R^2). A P value below 0.05 (shown in bold) indicates systematic differences in inferred genus composition between dried and frozen samples. Each test is based on 10 samples (5 dried, 5 frozen).

**Material**	**F-statistic**	**SES**	**P**	R^2
Bray-Curtis, non-rarefied				
feces 1	0.73	-0.62	0.67	0.035
feces 2	2.1	1.8	0.059	0.15
feces 3	0.74	-0.31	0.46	0.044
soil 1	5.2	5.4	**0.0066**	0.36
soil 2	2.6	1.3	0.079	0.13
soil 3	0.98	-0.12	0.28	0.043
Bray-Curtis, rarefied				
feces 1	1.06	0.019	0.36	0.072
feces 2	2.87	1.94	**0.042**	0.19
feces 3	5.36	5.17	**0.0058**	0.40
soil 1	8.26	7.65	**0.008**	0.58
soil 2	2.09	1.72	0.067	0.17
soil 3	3.61	5.59	**0.0082**	0.33
Jaccard, non-rarefied				
feces 1	3.91	1.35	0.091	0.045
feces 2	2.65	1.08	0.19	0.11
feces 3	1.44	-0.023	0.37	0.023
soil 1	6.62	5.60	**0.0057**	0.28
soil 2	5.21	3.12	**0.013**	0.18
soil 3	2.11	3.69	**0.004**	0.10
Jaccard, rarefied				
feces 1	0.96	-0.13	0.44	0.045
feces 2	1.57	0.57	0.21	0.065
feces 3	1.38	0.22	0.22	0.12
soil 1	4.59	6.50	**0.0054**	0.37
soil 2	2.29	2.49	**0.029**	0.18
soil 3	1.72	4.57	**0.0043**	0.18

Table S5: **PERMANOVA analysis, gene composition.** PERMANOVA test results, comparing weighted Bray-Curtis dissimilarities in gene (KO) composition between dried and frozen samples, separately for each material. Shown are the pseudo F-statistic, the standardized effect size (SES), the statistical significance (P) and the fraction of variance in pairwise distances explained by the treatment (R^2). A P value below 0.05 (shown in bold) indicates systematic differences in inferred gene composition between dried and frozen samples. Each test is based on 10 samples (5 dried, 5 frozen).

**Material**	**F-statistic**	**SES**	**P**	R^2
feces 1	0.79	-0.23	0.38	0.042
feces 2	9.3	6.9	**0.003**	0.44
feces 3	20.4	11.1	**0.002**	0.79
soil 1	16.8	10.7	**0.005**	0.80
soil 2	6.1	8.5	**0.003**	0.52
soil 3	7.0	8.8	**0.005**	0.59

Table S6: **PERMANOVA analysis, KEGG C composition.** PERMANOVA test results, comparing weighted Bray-Curtis dissimilarities in KEGG-C gene group composition between dried and frozen samples, separately for each material. Shown are the pseudo F-statistic, the standardized effect size (SES) and the statistical significance (P). A P value below 0.05 (shown in bold) indicates systematic differences in inferred gene group composition between dried and frozen samples. Each test is based on 10 samples (5 dried, 5 frozen).

**Material**	**F-statistic**	**SES**	**P**	R^2
feces 1	0.19	-0.42	0.85	0.012
feces 2	13.6	8.6	**0.003**	0.54
feces 3	46.2	12.9	**0.004**	0.87
soil 1	36.7	12.5	**0.004**	0.88
soil 2	11.4	9.9	**0.005**	0.69
soil 3	10.9	9.7	**0.001**	0.71

Table S7: **Location tests for KEGG C group abundances.** Overview of statistical location tests of KEGG C gene group proportions, i.e., comparing proportions of individual gene groups between treatments and separately for each material. Shown are the total number of gene groups considered, the number of groups exhibiting a statistically significant difference between the two treatments ($P < 0.05$), and the number of significant groups after Bonferroni correction. Each test is based on 10 samples (5 dried, 5 frozen).

**Material**	**Ngroups**	**Nsignificant**	**Nsignificant Bonferroni**
feces 1	487	7	0
feces 2	376	210	57
feces 3	306	250	120
soil 1	360	247	99
soil 2	364	165	41
soil 3	362	151	35

Table S8: **Overview of MAGs.** Number of MAGs constructed for each combination of material type and treatment. Also shown are the number of MAGs of at least medium quality (completeness ≥50%, contamination ≤10%).

Material type	treatment	NMAGs	Mean completeness (%)	Mean contamination (%)	NMAGs at least medium quality
feces	dried	135	55.5	6.34	61
feces	frozen	146	56.9	7.35	62
soil	dried	14	35.0	4.92	0
soil	frozen	7	26.4	1.90	0

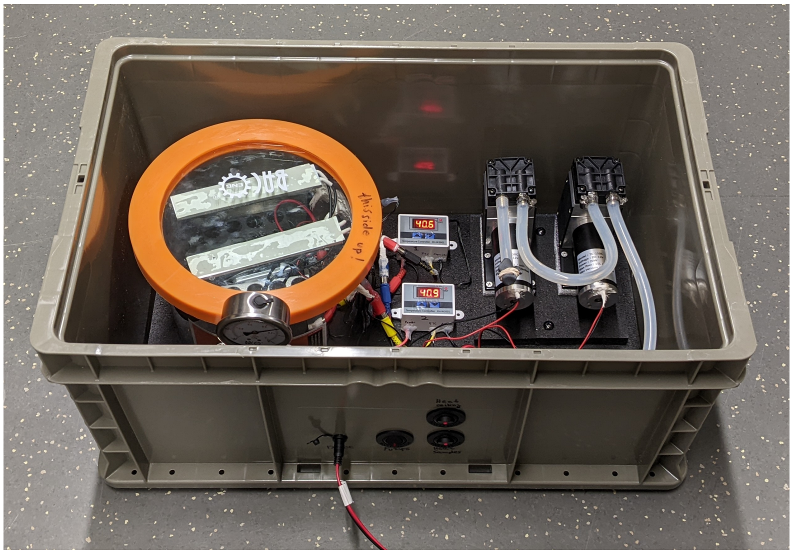

Figure S1: **Desiccator prototype.** Vacuum- and heat-assisted sample desiccator developed and used in this study.

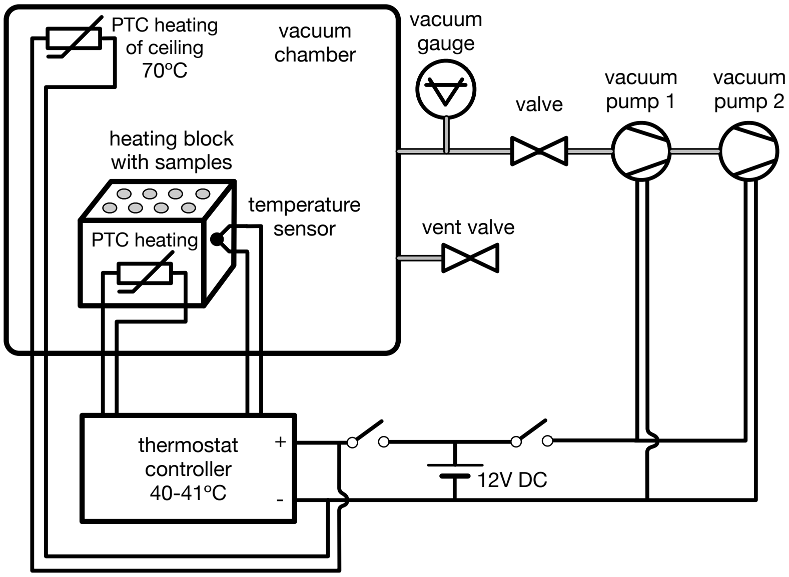

Figure S2: **Desiccator schematic.** Schematic diagram of the prototype desiccator developed and used in this study. Only one of two thermostat-controlled heating blocks is shown here for simplicity.

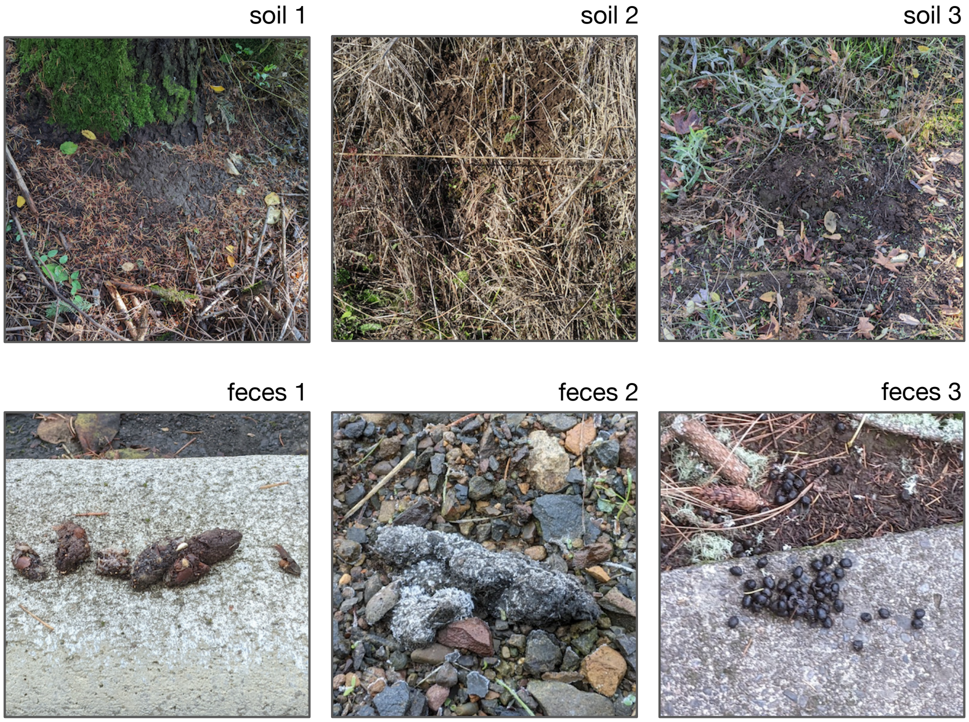

Figure S3: **Photos of samples prior to collection.** Photos of soil samples (top row) and fecal samples (bottom row) prior to collection.

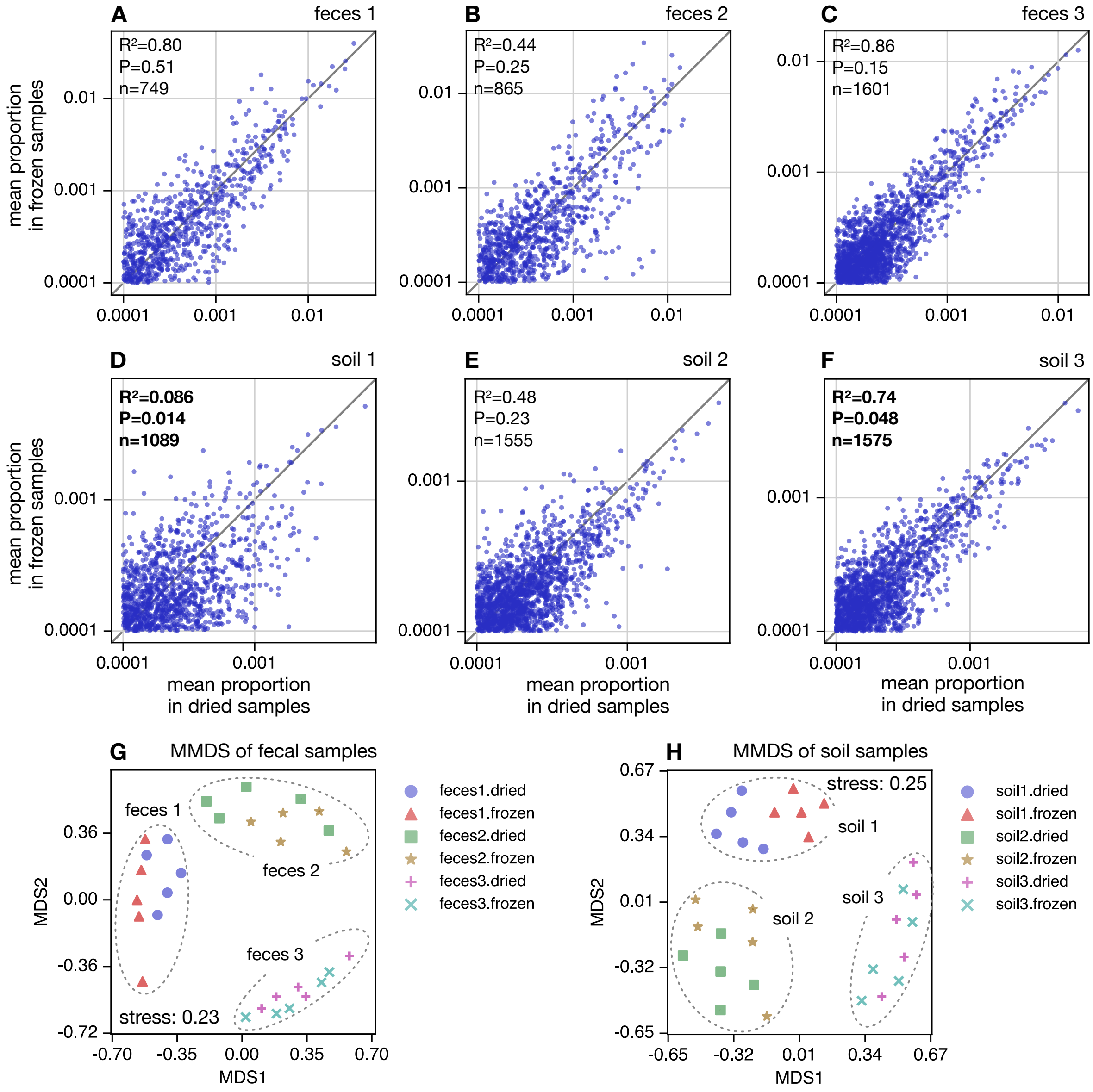

Figure S4: **ASV composition vs. treatment.** (A) Mean ASV proportions in dried fecal 1 samples (horizontal axis) compared to mean ASV proportions in frozen fecal 1 samples (vertical axis, one point per ASV). Averaging of proportions was done among the 5 replicates in each treatment. The diagonal is shown for reference. Inscriptions show the fraction of variance in the vertical axis explained by the horizontal axis (R^2), the number of ASVs considered (n), and the statistical significance of R^2 compared to a permutation null model under which ASV proportions are statistically indistinguishable in the two treatments (P). A significantly low R^2 (i.e., P<0.05) suggests that R^2 is lower than expected by chance, and that dried samples tend to yield different ASV proportions compared to frozen samples. (B–F) Similar to A, but for the remaining samples. Statistically significant R^2 values are boldened. (G) Metric multidimensional scaling plot of abundance-weighted Bray-Curtis dissimilarities between fecal samples, based on ASV proportions. Points correspond to samples, and are shaped and colored according to the material (feces 1–3) and treatment (dried vs frozen). The Kruskal stress is written in the plot. (H) Similar to G, but for soil samples. For similar plots using OTU or genus proportions see Figures 2 and S5, respectively.

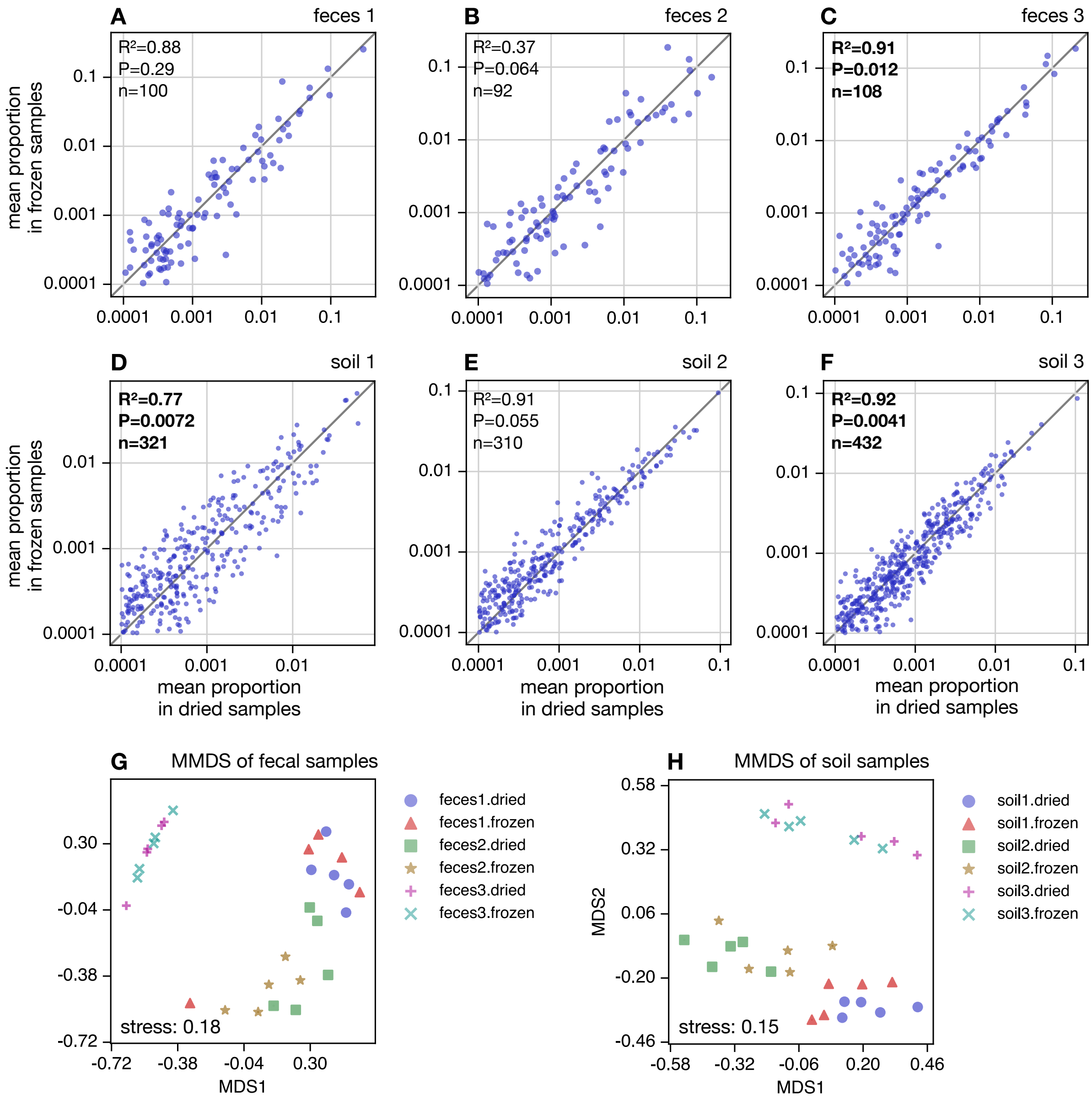

Figure S5: **Genus composition vs. treatment.** (A) Mean genus proportions in dried fecal 1 samples (horizontal axis) compared to mean genus proportions in frozen fecal 1 samples (vertical axis, one point per genus). Averaging of proportions was done among the 5 replicates in each treatment. The diagonal is shown for reference. Inscriptions show the fraction of variance in the vertical axis explained by the horizontal axis (R^2), the number of genera considered (n), and the statistical significance of R^2 compared to a permutation null model under which genus proportions are statistically indistinguishable in the two treatments (P). A significantly low R^2 (i.e., P<0.05) suggests that R^2 is lower than expected by chance, and that dried samples tend to yield different genus proportions compared to frozen samples. (B–F) Similar to A, but for the remaining samples. Statistically significant R^2 values are boldened. (G) Metric multidimensional scaling plot of abundance-weighted Bray-Curtis dissimilarities between fecal samples, based on genus proportions. Points correspond to samples, and are shaped and colored according to the material (feces 1–3) and treatment (dried vs frozen). The Kruskal stress is written in the plot. (H) Similar to G, but for soil samples. For similar plots using ASV or OTU proportions see Figures S4 and 2, respectively.

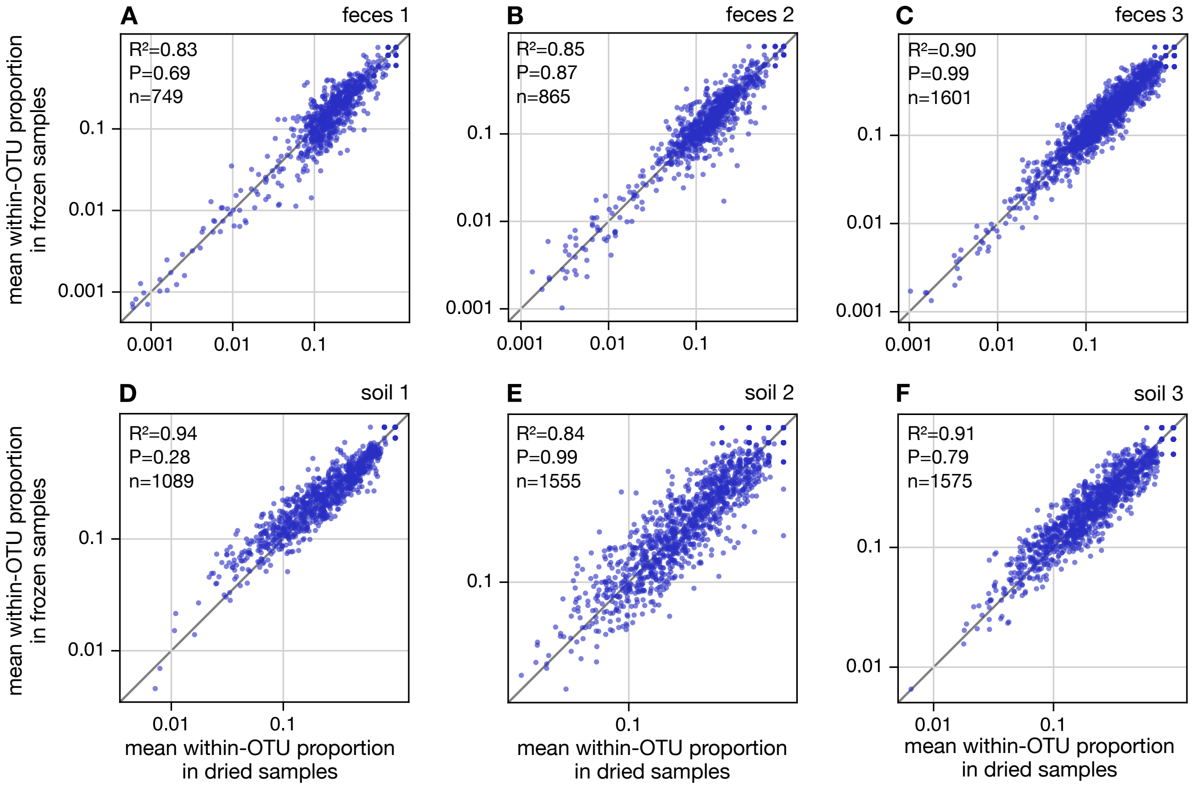

Figure S6: **ASV composition within OTUs vs. treatment.** (A) Mean ASV proportions within their respective OTU in dried fecal 1 samples (horizontal axis) compared to mean ASV proportions within their respective OTU in frozen fecal 1 samples (vertical axis, one point per ASV). Averaging of proportions was done among the 5 replicates in each treatment. The diagonal is shown for reference. Inscriptions show the fraction of variance in the vertical axis explained by the horizontal axis (R^2), the number of ASVs considered (n), and the statistical significance of R^2 compared to a permutation null model under which ASV proportions within their respective OTUs are statistically indistinguishable in the two treatments (P). A significantly low R^2 (i.e., P<0.05) suggests that R^2 is lower than expected by chance, and that dried samples tend to yield different ASV proportions compared to frozen samples. (B–F) Similar to A, but for the remaining samples. Statistically significant R^2 values are boldened.

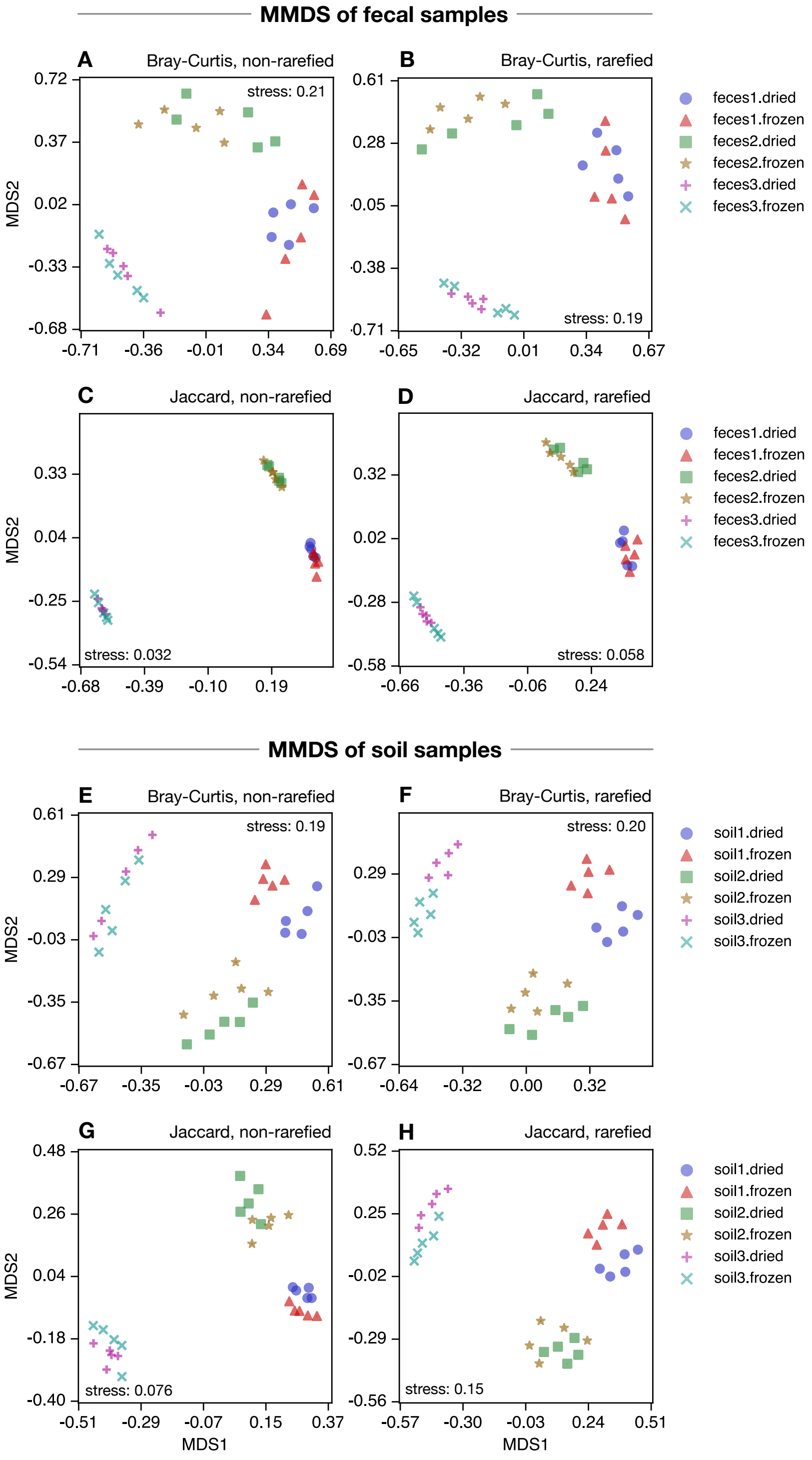

Figure S7: **Metric multidimensional scalings (OTU composition).** (A) Metric multidimensional scaling plot of abundance-weighted Bray-Curtis dissimilarities between fecal samples, based on OTU proportions. Points correspond to samples, and are shaped and colored according to the material (feces 1–3) and treatment (dried vs frozen). The Kruskal stress is written in the plot. (B) Similar to A, but based on a rarefied OTU table where all samples have equal numbers of reads. (C, D) Similar to A and B, but using abundance-weighted Jaccard dissimilarities. (E–H) Similar to A–D, but focusing on soil samples. For analogous PCoA plots see Fig. S12.

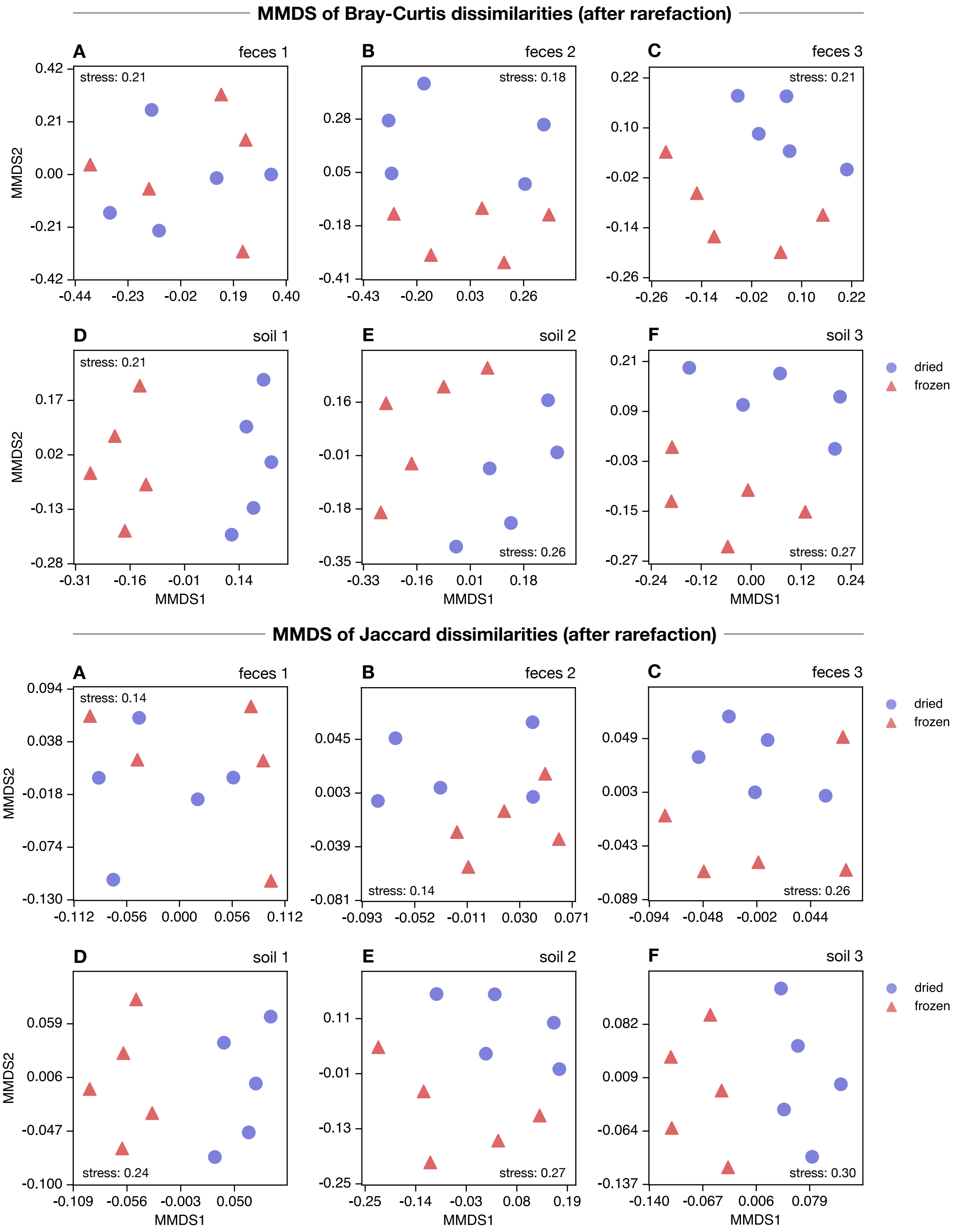

Figure S8: **Metric multidimensional scalings, separately for each material (OTU composition).** (A) Metric multidimensional scaling plot of abundance-weighted Bray-Curtis dissimilarities between "feces 1" samples, based on OTU proportions after rarefaction. Points correspond to samples derived from feces 1 material, and are shaped and colored according to treatment (dried vs frozen). The Kruskal stress is written in the plot. (B–F) Similar to A, but for feces 2–3 and soils 1–3. For PERMANOVA tests quantitatively examining the separation by treatment see Table S3.

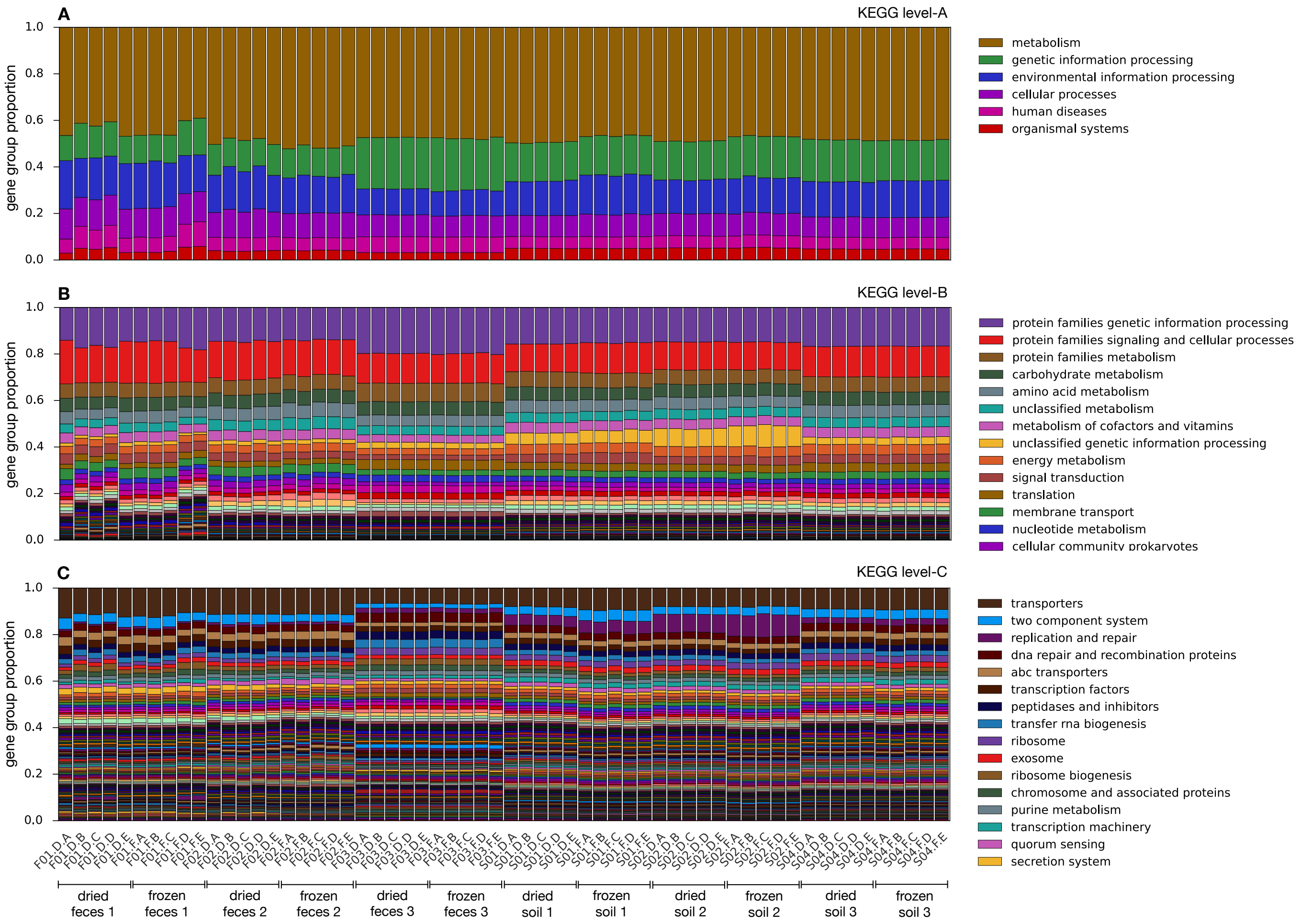

Figure S9: **KEGG gene group profiles.** (A) Estimated proportions of KEGG-level-A gene groups in each sample. Each column corresponds to a sample, and bar segment heights correspond to group proportions, which are based on numbers of reads mapped. (B, C) Similar to A, but showing KEGG level-B and KEGG level-C groups, respectively. In B and C, inly the top 100 groups are shown and only a subset of these is included in the legend to avoid cluttering the figure.

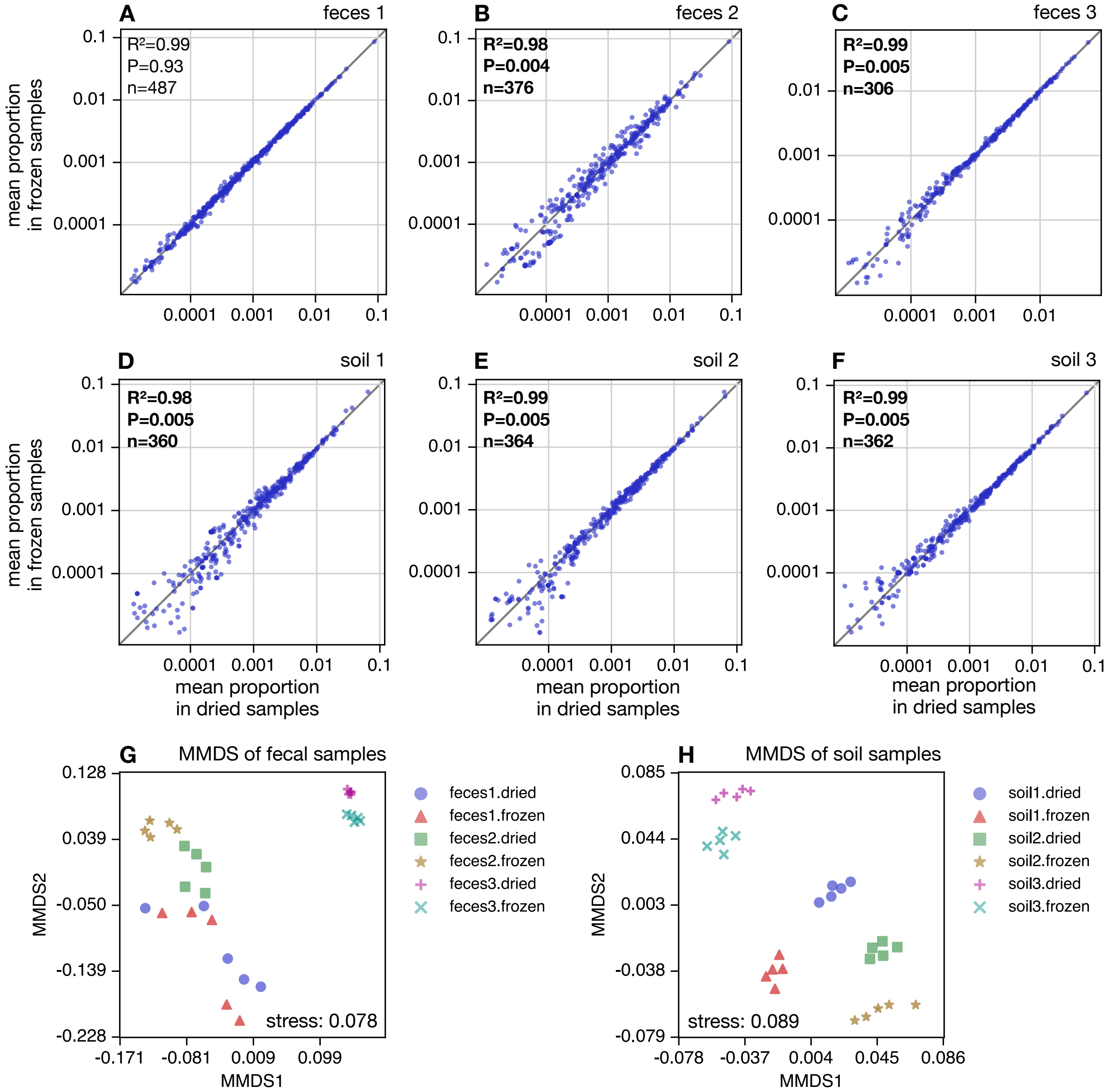

Figure S10: **KEGG C composition vs. treatment.** (A) Mean KEGG C gene group proportions in dried fecal 1 samples (horizontal axis) compared to mean gene group proportions in frozen fecal 1 samples (vertical axis, one point per gene group). Averaging of proportions was done among the 5 replicates in each treatment. The diagonal is shown for reference. Inscriptions show the fraction of variance in the vertical axis explained by the horizontal axis (R^2), the number of gene groups considered (n), and the statistical significance of R^2 compared to a permutation null model under which gene group proportions are statistically indistinguishable in the two treatments (P). A significantly low R^2 (i.e., P<0.05) suggests dried samples tend to yield different gene group proportions compared to frozen samples. (B–F) Similar to A, but for the remaining samples. Statistically significant R^2 values are boldened. (G) Metric multidimensional scaling plot of weighted Bray-Curtis dissimilarities between fecal samples, based on gene group proportions. Points correspond to samples, and are shaped and colored according to the material (feces 1–3) and treatment (dried vs frozen). The Kruskal stress is written in the plot. (H) Similar to G, but for soil samples. For similar plots using gene (KO) proportions see Fig. 3.

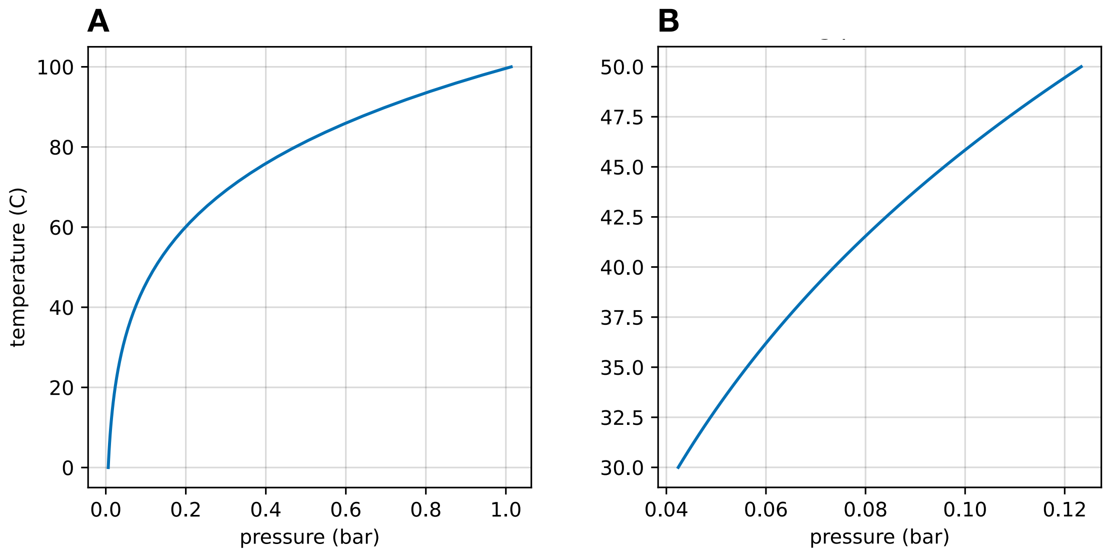

Figure S11: **Boiling point of water.** (A) Boiling temperature of water at pressures 0–1 bar. (B) Boiling temperature of within a narrower pressure range, covering temperatures 30–50°C. Based on the empirical formula in (Thomson, 1946, Eq. 29).

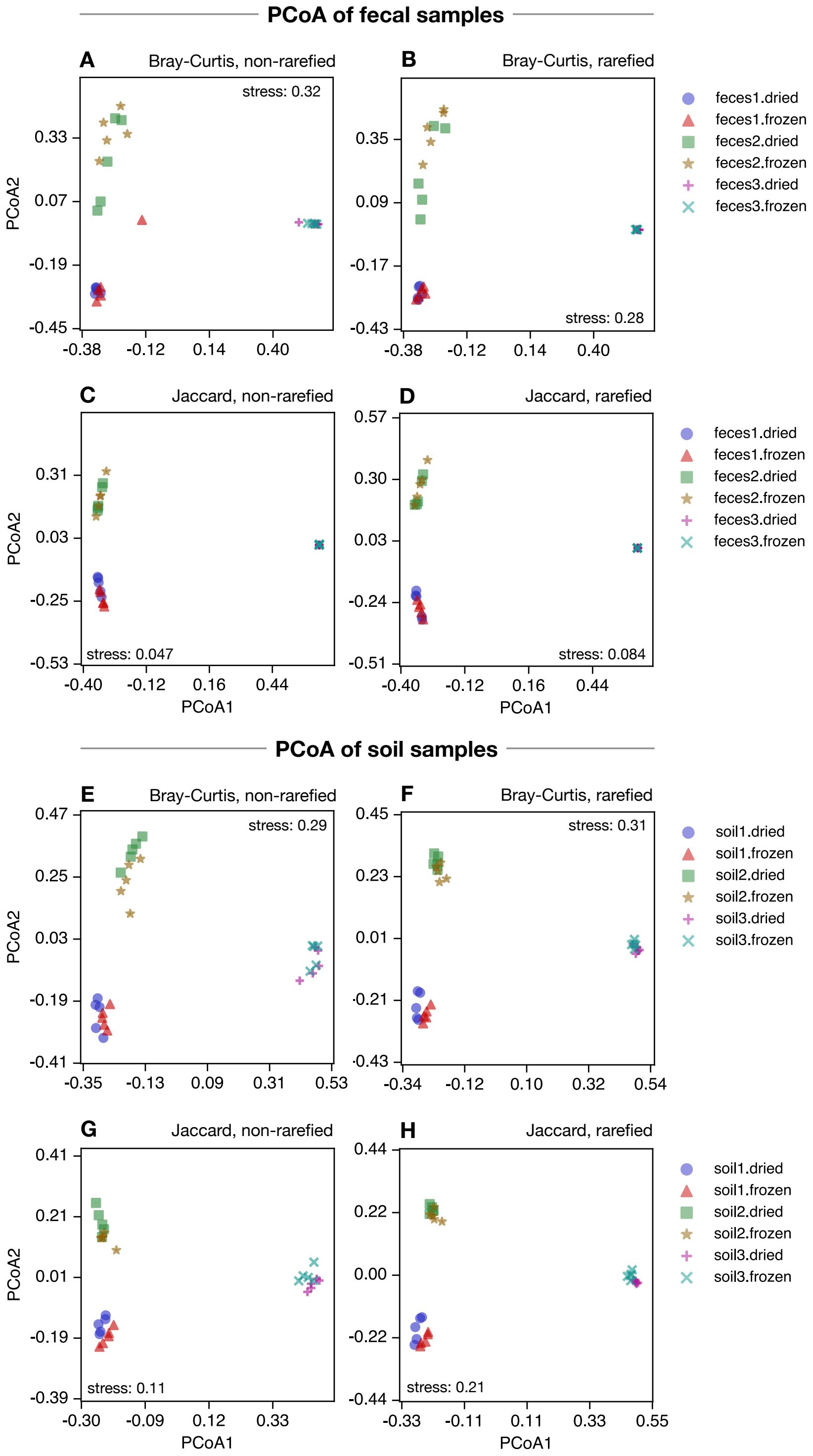

Figure S12: **PCoA (OTU composition).** (A) Principal coordinates analysis (PCoA, aka. classical multidimensional scaling) of abundance-weighted Bray-Curtis dissimilarities between fecal samples, based on OTU proportions. Points correspond to samples, and are shaped and colored according to the material (feces 1–3) and treatment (dried vs frozen). The Kruskal stress is written in the plot. (B) Similar to A, but based on a rarefied OTU table where all samples have equal numbers of reads. (C, D) Similar to A and B, but using abundance-weighted Jaccard dissimilarities. (E–H) Similar to A–D, but focusing on soil samples. For analogous metric multidimensional scaling plots see Fig. S7.

References

Thomson, G.W. (1946) The Antoine equation for vapor-pressure data. *Chemical Reviews*, 38, 1–39.
